# Emergency Department Clinical Quality Registries: A Scoping Review

**DOI:** 10.3390/healthcare13091022

**Published:** 2025-04-29

**Authors:** Viet Tran, Giles Barrington, Simone Page

**Affiliations:** 1 Royal Hobart Hospital, Tasmanian Health Service, Hobart 7000, Australia; 2 Tasmanian School of Medicine, University of Tasmania, Hobart 7000, Australia; 3 Menzies Institute for Medical Research, University of Tasmania, Hobart 7000, Australia; 4 Tasmanian Emergency Medicine Research Institute, Hobart 7000, Australia

**Keywords:** emergency department, emergency medicine, registries, clinical quality registry, safety, quality

## Abstract

Background: Emergency departments (ED) are vital within the health system, often representing the first hospital contact for patients who are undifferentiated and may be critically ill. Although advancements in digital technology and increasing use of electronic medical records in health systems have led to the dramatic growth of large data sets, the presence of ED clinical registries to measure quality of care in the literature is currently unknown. Objectives: Our scoping review aims to investigate the extent of emergency department clinical registries reported in peer-reviewed literature. Methods: We conducted a scoping review of ED registries in accordance with the PRISMA-ScR checklist. Searches were undertaken in PUBMED, EMBASE, and SCOPUS. Studies were included if they described a clinical registry with a focus on the ED. Results: We identified 60 manuscripts with 27 identified as primary registries (6 had a general scope, 21 were condition or population specific). The remaining 33 papers were investigational reports sourced from the identified primary registries. Funding sources were identified for some registries: three by research grants, two by medical colleges, five by government organizations or initiatives, two by pharmaceutical companies, and three by research institutes. No funding information was provided in 12 studies. The reported registry periods ranged from 31 days to 4018 days (median 365 days, IQR 181–1309 days). A grey literature search revealed that six registries were ongoing. Conclusions: Internationally, there appears to be a wide degree of heterogeneity with primary ED registry publications and secondary publications. Having a standardized approach to ED registries is needed. Integrating ED registries with a learning health system model will enable clinicians to serve their community proactively and with a focus on quality, rather than the current safety-focused approach.

## 1. Introduction

Emergency departments (EDs) play a vital role within the health system, often representing the first point of hospital contact for patients who are undifferentiated and may be critically ill [1]. The ED manages a high volume of patients who range in acuity from the critically ill through to the worried well. This places a significant burden on ensuring timely diagnosis and management to avoid potentially avoidable adverse outcomes from care [2]. The ED is therefore a patient care environment that is highly vulnerable to the provision of low-quality care and breaches in patient safety [3]. The exponential growth of ED overcrowding creates further challenges to delivering standardized, high-quality care [4].

A variety of solutions have been developed to try and counter these challenges, including guidelines, policies, and pathways. Despite these best intentions, EDs remain significantly unsafe [5]. In recent years, advancements in digital technology and increasing use of electronic medical records in health systems have led to dramatic growth in large clinical data sets. Harnessing these data to improve health care has taken on many forms and defined in different ways to include databases, audits, and registries. These registries, along with databases and audits, provide a comprehensive framework for tracking patient outcomes, identifying best practices, and facilitating evidence-based decision-making. By harnessing this wealth of information, healthcare providers can pinpoint areas for improvement, tailor treatments to individual patient needs, and ultimately deliver more effective and personalized care. Patient registries can be defined as organized systems that use observational study methods to collect uniform data (clinical and other) to evaluate specified outcomes for a population defined by a particular disease, condition, or exposure, and that serve a predetermined scientific, clinical, or policy purpose(s) [6]. Audits on the other hand can be seen as one-off collections of data.

Clinical quality registries (CQRs) have emerged as a necessary approach to systematically collect, analyze, and report information about the care and outcomes being delivered by health service organizations, and serve as a fundamental driver for ongoing improvements in the delivery of safe and high-quality care [7]. Mature CQRs such as the Australian and New Zealand Intensive Care Society Centre for Outcomes and Resources Evaluation (ANZICS CORE) have the capability to also shape health policy, integrate data through linkage projects, and inform clinical practice in real time whilst also providing infrastructure for measuring the translation of evidence into practice [8]. For emerging areas of health care, CQRs have been shown to be a catalyst to create clinical evidence for the development of best practice guidelines such as the Burns Registry of Australia and New Zealand [9]. Since care for many diseases and illnesses as well as the utilization of specific healthcare resources, such as trauma teams, occur within the ED, the data reflecting the ED phase of care are being collected for these related CQRs. However, given the breadth and varied governance of these CQRs, ED staff often have little input into their methodology, maintenance, analysis, and interpretation [2].

Clinical quality registries can be categorized across three broad areas: (1) procedures, devices, or drugs, (2) disease or illness, or (3) specific healthcare resource (Table 1) [10]. Although there is no international repository for CQRs, country-level repositories do exist. The National Institutes of Health have a list of 81 registries, and the Australian Commission on Safety and Quality in Health Care have developed the Australian Register of Clinical Registries, which currently lists 125 CQRs [11,12].

Each CQR category presents their own challenges for creating a registry. Potential barriers to constructing an informed ED registry include determining key quality outcome measures. On a more technical level, the volume and heterogeneity of patients and potential data points for collection, rapid turnover, heterogeneous data handling, storage and retrieval, rigorous national and state data privacy laws, and institutional and jurisdictional requirements for data sharing agreements pose significant challenges to creating an ED registry [18]. Finally, the financial outlay for the significant infrastructure required to support an ED CQR can be a barrier.

To our knowledge, a review of ED-focused CQRs has not been published. Our scoping review aims to investigate the breadth of emergency department (ED) clinical registries documented in peer-reviewed literature.

## 2. Methods

This scoping review was guided by the Joanna Briggs Institute methodology for scoping reviews and in compliance with the Preferred Reporting Items for Systematic reviews and Meta-Analyses extension for Scoping Reviews (PRISMA-ScR) recommendations (Supplementary Table S1) [19,20]. The search protocol was developed by VT, GB, and SP and objectives, search methods, and registry inclusion criteria were prespecified before the commencement of the study. This study did not require approval by an institutional ethical review board as no individual patient data were included and data regarding registries were publicly available. Preregistration of the study protocol was published in Inplasy (INPLASY202440119—https://inplasy.com/inplasy-2024-4-0119 accessed on 29 April 2024) prior to commencement.

### 2.1. Search Strategy

The search strategy (Appendix A) was developed in accordance with the Peer Review of Electronic Search Strategies (PRESS) criteria [21]. The following electronic databases were searched: National Library of Medicine via PubMed, Embase, and Web of Science and were chosen as they collectively provide comprehensive coverage of biomedical literature, ensuring a thorough and diverse range of relevant studies [22]. The search period included date of database inception to April 2024.

The results of the database searches were screened using the web-based reviewing platform Covidence^TM^ (Veritas Health Innovation, Melbourne, Australia). Duplicate studies were removed. Title and abstract screening were performed independently by authors VT and GB where suitability for inclusion was assessed against the eligibility criteria. Following title and abstract screening, identified publications were retrieved and imported to Covidence^TM^ for full text review. Full-text screening of all included publications was performed by authors VT and GB independently. Any disagreements between reviewers during screening of title and abstract or full text were resolved through discussion with a third review author (SP) on the study team.

For publications where it became apparent that they were referencing a registry, a search for the registry occurred using the name of the registry in the PUBMED database to identify the primary publication for that registry.

### 2.2. Eligibility Criteria

#### 2.2.1. Inclusion Criteria

Full-text articles published in English describing ED registries were included. We defined an ED registry as a systematic data collection program (using a database, databank, or register) for monitoring standardized indicators of care quality (including safety) where ED care was the focus. Our inclusion and exclusion criteria only considered registries where the ED led the registry setup and maintenance. By having the ED as the lead department, it ensures that the most appropriate metrics are being measured to answer questions fundamental to the quality of care in the ED. Furthermore, ED staff will have the greatest understanding of the data, its interpretation, and, most importantly, its limitations [18]. Publications were included from protocol publication through to secondary analysis of registry data. Single-center, multicenter, regional, statewide, national, and multinational registries were also included.

#### 2.2.2. Exclusion Criteria

Publications were excluded if they were developed for epidemiological disease monitoring without collection of clinical care indicators. Publications were also excluded if they were purely for administrative, system monitoring, or financial purposes. Publications where the primary locus of care was not in the ED were excluded. Publications that were case reports, case series, narrative reviews, editorials, short communications, case studies, or conference abstracts were also excluded. Non-human studies were also excluded.

### 2.3. Data Extraction and Analysis

Data were extracted from included papers by authors VT and SP. Uncertainties or discrepancies were resolved through discussion with a third review author (GB). The data extraction template included funding source, trial number, type of registry, target demographic, population of interest, start and end dates for the registry, inclusion and exclusion criteria, data sources, collection tools, data entry procedures, number of participants included in the registry, availability of a data dictionary and data sharing arrangements, country of origin, number of participating Eds, and ethical approvals.

Registries identified were categorized based on their scope as well as on the sequence in which they were published. For scope, ED registries were described as ***general*** if the inclusion criteria included all ED presentations and conditions, or ***condition or population specific*** if the data captured were restricted. For studies that identified an ED registry within the search strategy more than once, the oldest paper was considered the ***primary registry publication*** and subsequent papers as ***secondary publications***.

Aims, objectives, results, and conclusions were also extracted from publications to understand how registry data are being used. Included studies were not appraised for methodological quality or risk of bias as this is not customary for scoping reviews.

Data analysis included developing summary statistics and frequency counts. SP developed summary tables.

## 3. Results

### 3.1. Search Results

**Figure 1:**
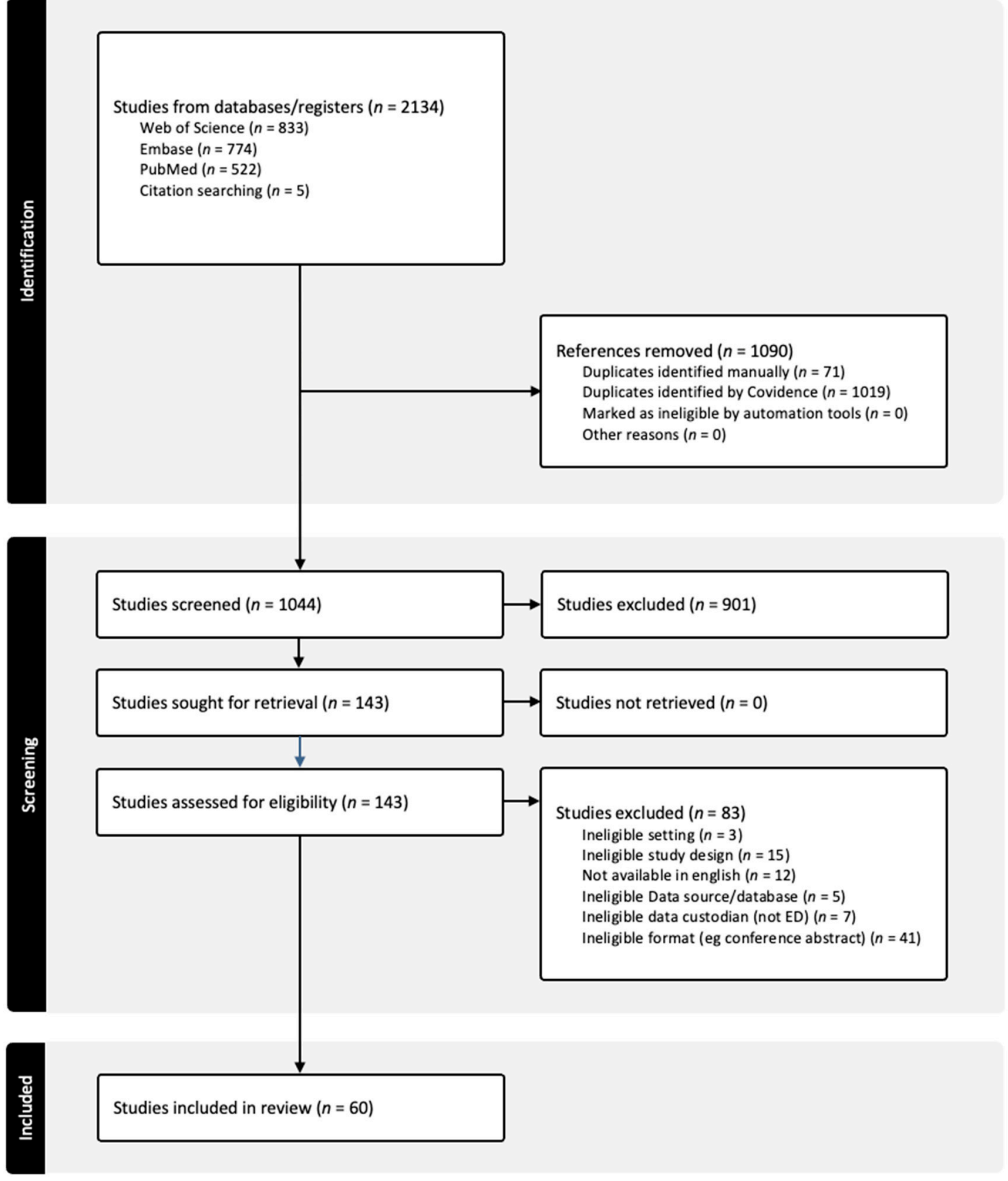
PRISMA diagram summarizing the search strategy used to identify emergency department clinical quality registries meeting inclusion criteria.

The initial literature search identified 2134 publications (Figure 1). After removal of 1090 duplicates, 901 studies were not eligible based on title and abstract screening by two independent reviewers (VT and GB). Of the 143 full-text publications acquired and screened independently by VT and GB, a further 83 publications were excluded due to a variety of reasons described in the PRISMA flow diagram (Figure 1), such as ineligible format (conference abstract) or ineligible data custodian (not an ED). As a result, 60 studies were eligible for review (Table 2) [23,24,25,26,27,28,29,30,31,32,33,34,35,36,37,38,39,40,41,42,43,44,45,46,47,48,49,50,51,52,53,54,55,56,57,58,59,60,61,62,63,64,65,66,67,68,69,70,71,72,73,74,75,76,77,78,79,80,81,82,83]. Notably, there were six general registries and six airway-specific registries with few secondary publications from general registries.

### 3.2. Characteristics of Primary ED Registry Publications

**Figure 2:**
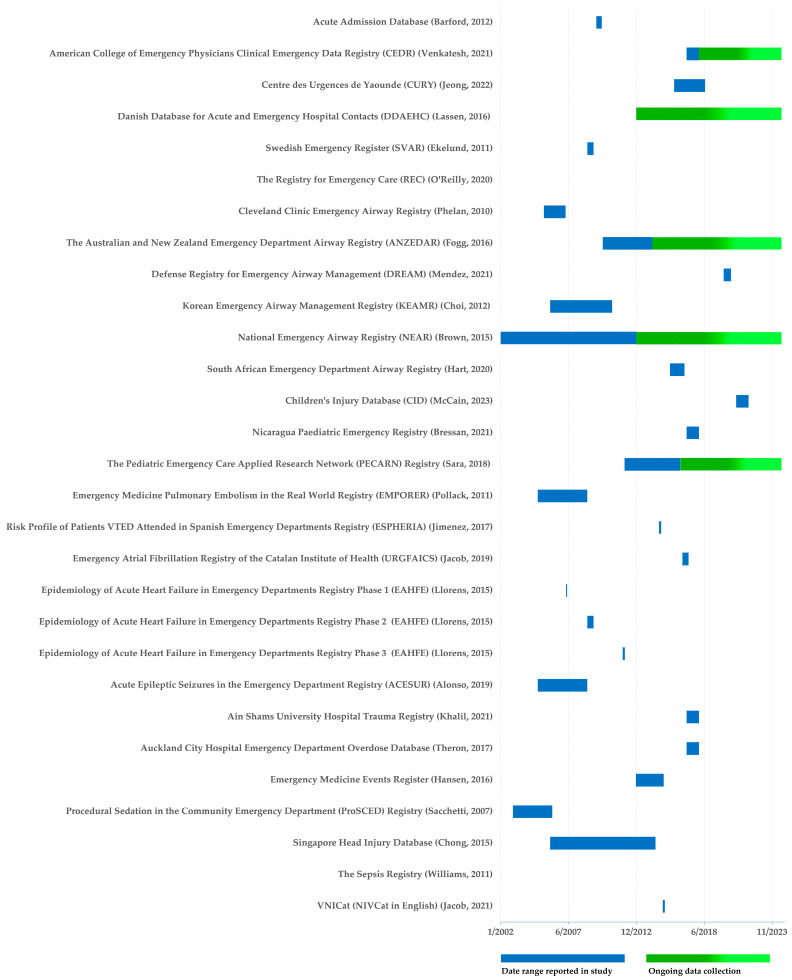
Timeframes for primary ED registry publications [23,24,27,30,33,34,35,39,43,44,45,47,48,49,51,53,54,55,56,57,58,60,62,63,65,71,74,76].

Our scoping review yielded 27 registries that fit the inclusion criteria for a primary ED registry publication (Table 2). Six of these registries were general ED registries. Three registries were specific to the same population, namely pediatrics. The remaining 18 registries were specific to a condition. Airways was the most common condition with six registries, followed by two registries that covered different aspects of venous thromboembolism. The remaining ED registries addressed independent conditions. The number of EDs involved in each registry varied from 1 to 889 (median 5.5, IQR 1–14). Country representation included a total of 13 countries with USA the most frequently contributing with eight registries (two general, six specific) followed by Australia with five (one general, four specific), Denmark two (two general, zero specific), Spain four (zero general, four specific), and New Zealand two (zero general, two specific). The reported registry periods ranged from 31 days to 4018 days (median 365 days, IQR 181–1309 days). Following a grey literature search of the 27 primary ED registry publications, 6 were found to be ongoing; 3 general, 2 airway-specific, and 1 pediatric-specific. Distribution of these registries over time is shown in Figure 2.

### 3.3. Funding for Primary ED Registry Publications

For registries where funding was acknowledged, three were funded by research grants, two by medical colleges, five by government organizations or initiatives, two by pharmaceutical companies, and three by research institutes. For registries identified as ongoing (Figure 2), sources of funding were identified as medical college for one registry, government organization or initiative for three registries, research grants for one registry, and no funding acknowledged for one registry. Twelve of the twenty-seven primary ED registry publications did not acknowledge any funding for the registries described. Funding amounts were not described for any of the studies.

### 3.4. Aims, Results, and Conclusion in Primary ED Registry Publications with a General Scope

Aims, results, and conclusions for primary ED registry publications with a general scope provided insight into the breadth of each manuscript (Table 3). Three publications focused on registry methodology, feasibility and evaluation, while two described its use for performance measurements and one described its use for measuring the quality of clinical care.

For publications that focused on registry methodology, feasibility, and evaluation, 2 concluded that creating a registry and evaluating a pilot project was feasible, with the other describing methodology without acknowledging feasibility. One paper also described a desire to expand the dataset to include non-ED metrics, another described the potential for use in quality improvement, and the last described expansion to additional EDs.

Lassen et al. [48] was the only general primary ED registry publication that described the evaluation of quality of clinical care. Notably, this registry included specific outcomes and process health care quality indicators. Performance measures in the ED are often represented by time-based targets including length of time in the ED and time awaiting medical review. Two general ED registries described the use of a registry to inform these performance measures, although one paper was simply the protocol.

### 3.5. Aims, Results and Conclusion in Primary ED Registry Publications Specific for a Condition or Population

Aims, results, and conclusions for primary ED registry publications specific for a condition or population provided insight into the scope of each manuscript (Supplementary Table S2). Unlike general scope primary ED registry publications, condition-specific primary ED registry publications focused on clinical quality including evaluating or benchmarking against standards. The only population-specific registries were for pediatrics, and these were similar to general scope primary ED registry publications focusing on registry methodology, feasibility, and evaluation.

### 3.6. Aims, Results and Conclusion in Secondary Publications

Of the 27 primary ED registry publications, 16 did not have a secondary study (Table 3). Of those that did have secondary publications, the median number of studies was 1 (IQR 1–2). Two general ED registries each had 1 secondary publication while 24 secondary publications were identified for all airway-specific registries with the NEAR registry accompanied by 19 associated studies (Table 4).

Aims, results, and conclusions for secondary publications were similar to their primary ED registry publications (Supplementary Table S3). Secondary publications with a focus on specific conditions reported on clinical quality through a variety of means, including benchmarking and describing trends in practice. Secondary publications with a general scope reported on performance indicators in the form of time-based performance.

## 4. Discussion

Emergency departments are often tasked with managing a high proportion of undifferentiated patients who range from the critically ill through to the worried well [2]. This combination of undifferentiated and unwell patients places a significant burden on ensuring timely diagnosis and management to avoid significant adverse outcomes from care [2]. Clinical registries are focused on the quality of health care within specific clinical domains by systematically analyzing health-related data for an eligible population and serves as an efficient approach to the assessment of quality care in the ED setting [7]. This scoping review sought to understand the current use of ED registries.

### 4.1. Emergency Department Registries Reported in the Literature

Our review identified 27 primary ED registry publications, representing a diverse range of focuses and geographical representations. Notably, most registries were condition-specific, with airway being by far the most common condition studied (33.3% of all condition-specific primary ED registry publications). This may represent the fact that airway management in the ED setting is of critical importance [84]. It may also be that there are more reproducible clinical quality measures within the process of airway management in this setting [84]. The reality is likely to be multifactorial but suggests that airway registries may be more feasible at a local level and therefore individual EDs or networks of EDs could consider establishing or contributing to an ED airway registry to pilot the creation of a registry in the ED. This process would assist in understanding the technical requirements as well as local ethical, governance, and data sharing requirements prior to embarking on a larger registry.

Whereas condition- or population-specific ED registries offer an easier approach to understanding the infrastructure needs for local registry set up and maintenance, general ED registries serve a broader purpose given the general nature of emergency medicine as a specialty and the need to interrogate many aspects of this heterogenous patient population. The difference between condition- or population-specific registries and general registries is therefore vastly different and requires a significantly higher investment both financially and administratively. All the general ED registries included in our search strategy utilized a largely administrative data set. This seems to be the most practical method for developing general ED registries, though it is limited primarily to performance data, particularly time-based targets. A natural evolution should involve the inclusion of clinical data to shift the analysis to clinical care and patient-centered outcomes. How clinical data is linked to administrative data and what clinical data should be prioritized for capture are all considerations that will require further research and consensus with the involvement of consumers and policymakers alike [18].

### 4.2. Emergency Department Registry Scope

The analysis of primary ED registry publications specific to conditions or populations revealed distinct trends and focuses compared to general scope primary ED registry publications. Condition-specific registries predominantly emphasized clinical quality, often through evaluation and benchmarking against established standards. This focus on clinical quality underscores the importance of these registries in enhancing patient care and outcomes by providing a framework for continuous improvement and adherence to best practices.

In contrast, the only population-specific registries identified were those targeting pediatric populations. These registries shared similarities with general scope primary ED registry publications, particularly in their emphasis on registry methodology, feasibility, and evaluation. This alignment suggests that while the target population may differ, the foundational principles guiding the development and implementation of these registries remain consistent.

Our scoping review describes a largely heterogenous set of ED registries. As a result of this, quality indicators were not well described. To enhance the selection of quality indicators, it would be advantageous to adopt a stakeholder-driven approach. This approach should prioritize the values of patients and communities, as well as the actionable improvement priorities identified by healthcare providers. Additionally, there should be a greater emphasis on measuring distinct and discernible processes of care [85].

### 4.3. Temporal Scope of Emergency Department Registries

Most registries were time-limited, with a median duration of one year (0.05–3.59 years). A grey search of all primary ED registry publications recognized six of these registries as ongoing, three of which are general ED registries. This time limitation may exist for a variety of reasons. It may reflect the challenges associated with sustaining long-term registry efforts, including funding, data collection, and maintenance. A more plausible explanation is that researchers use the term registry interchangeably with traditional data collection tools for audit or research and databases that do not meet the criteria of a registry [6].

### 4.4. Funding Source for Emergency Department Registries

Health economic studies have demonstrated that relatively small injections of funding to supplement existing efforts at creating and maintaining a clinical registry is likely to be highly cost effective [86,87]. The funding sources described for the registries identified were varied, ranging from hospital-based funding to government organizations and pharmaceutical companies. Nearly half of the registries did not acknowledge any funding, which raises questions about sustainability and potential biases in the data collection processes. For the registries that did report funding, government organizations and research institutes were the most common sources. Of all registries that were ongoing, only three of the five had reported funding, which suggests the role of funding may only be one part of a successful registry.

None of the studies reported the amount of funding provided for either registry-related costs or study-related costs. This would have been useful to provide an indication for those wanting to initiate a registry to better understand the financial requirements and align funder expectations. Future studies should aim to provide more transparency in funding to better understand the resources required for developing, maintaining, and interrogating registry data.

### 4.5. ED Registries as a Catalyst for Further Publications

The examination of secondary publications reveals the impact and scope of these registries. Of the 27 primary ED registry publications analyzed, 16 did not have any secondary publications, highlighting a potential area for future research and exploration. Some non-ED registries such as the Australian Trauma Registry have been successful with more abundant reporting with over 14 secondary publications since the registry started in 2012 [88]. One hypothesis includes the lack of ongoing funding to support such endeavors. For those registries with secondary publications, the median number of studies was relatively low, with a median of 1 (IQR 1–2). This finding suggests that while some registries are actively contributing to the research landscape, there is significant room for expansion and the barriers for these should be explored.

Interestingly, the NEAR registry, an airway-specific registry, stood out with 19 secondary publications, indicating a robust research output and a strong focus on airway management within the ED context. This contrasts with the general ED registries, which had fewer secondary publications. The secondary publications for condition-specific registries primarily reported on clinical quality through various means, including benchmarking and trend analysis.

### 4.6. ED Registries as a Catalyst for Quality—A Piece of the Learning Health System Puzzle?

Registries themselves serve as a rich source of data and on their own serve no other purpose. It is up to policy makers, ED administrators, and clinicians to utilize these data to deliver these quality initiatives. Although quality and safety are often used in the same phrase, the current driver for enhancing care prioritizes the delivery of safe (rather than quality) care. In the context of resource-poor services, the result of this focus is a system that is reactive without the opportunity or resources to focus on quality. Learning health systems (LHSs) have emerged as a popular concept to bring focus back on quality through a standardized framework [89]. The Institute of Medicine coined the term Learning Health System (LHS) in 2007, describing it as a health system where *“science, informatics, incentives, and culture are aligned for continuous improvement and innovation, with best practices seamlessly embedded in the care process, patients and families active participants in all elements, and new knowledge captured as an integral by-product of the care experience”* [89]. Although there is debate around a specific definition for an LHS, a review of bibliometric trends for LHSs found a large degree of convergence describing LHSs as *‘achieving healthcare quality improvement by using big data and embedding data analysis and intelligent decision-making into routine care delivery processes’* [90]. A fundamental requirement for this system to work, and indeed any quality improvement strategy, relies on the availability of data relevant to the quality being evaluated. The appeal of registries in the ED setting is that quality can be truly assessed on a broad level compared with current ad hoc strategies that realistically result in no evaluation and therefore limited understanding of the true quality of care being delivered.

## 5. Limitations

There are several limitations to this review. The search strategy used terms in English. Limiting our scoping review to only English language papers can introduce language bias and geographic bias, potentially excluding relevant studies published in other languages and thereby affecting the comprehensiveness and generalizability of the review’s findings. Future reviews should consider the inclusion of non-English papers. Our search strategy was limited to publications and therefore excludes registries that were operationalized without any publications (e.g., local quality improvement or annual reports not published in the peer review literature). We retrospectively searched the grey literature where a registry that fulfilled the inclusion criteria was identified but was not the primary paper. One example of this is the World Health Organization’s clinical registry tool, a web-based platform for aggregation and analysis of case-based data from emergency care visits [91]. Most health service quality improvement projects and evaluations are contained within the service itself and therefore initiatives generated from registry data are not able to be quantified unless published in the peer review literature.

Some countries adopted a whole health system approach for clinical quality registries based around innovative implementation of health information management technology. Emergency department subsets of these large registries were excluded from our search strategy as they were not considered a feasible approach for many clinicians and clinician researchers considering developing and implementing an ED registry.

Finally, the practical implications of developing ED registries were unable to be explored given the paucity and variable literature available. A more focused area of ED registry analysis utilizing a systematic review methodology in the future should be undertaken to provide these insights.

## 6. Conclusions

The use of clinical registries for quality improvement and research has grown significantly and will continue to do so with advancements in information technology. Whilst many registries are used to evaluate and enhance the quality of care provided, the chaotic nature of EDs has been overlooked. From our scoping review, internationally there appears to be a lack of published registries and secondary publications. Of those that do exist, ED-based airway registries have been the most abundant and can potentially serve as a ‘pilot’ registry for departments to understand capacity and capability for managing and using registry data. Funding appears to be a primary barrier to success. The establishment of comprehensive general ED registries presents an opportunity to evaluate care quality. By integrating these registries within a learning health system framework, clinicians can adopt a proactive, quality-centered approach to community healthcare, moving beyond the current reactive and safety-oriented model.

## Figures and Tables

**Table 1:** Categories and examples of clinical quality registries.

Category	Scope	Clinical Quality Registry Example
Procedure, device, or drug	Joint replacement	The American Joint Replacement Registry [13]
	Ventricular Assisted Device	The Spanish Registry of Durable Ventricular Assist Devices [14]
Disease or illness	Hip fracture	The Swedish Fracture Register [15]
	Stroke	The Australian Stroke Clinical Registry [16]
Specific healthcare resource	Trauma	The Australian Trauma Registry [17]
	Intensive Care	The Australian and New Zealand Intensive Care Society Centre for Outcomes and Resources Evaluation [8]

**Table 2:** Count of ED clinical quality registries by type and scope of registry.

Scope	Primary Registry Publications ^1^	Secondary Publications ^2^
General	6	2
Condition or population specific	21	31

^1^ The oldest paper where a registry was identified more than once. ^2^ Subsequent paper(s) where a registry was identified more than once.

**Table 3:** Aims, results, and conclusion of primary ED registry publications.

Registry		Aim	Results	Conclusion
Acute Admission Database	Barford, 2012 [74]	The objective of this article is to (1) describe the formation and design of the ‘Acute Admission Database’ and (2) characterize the cohort included.	In primary triage, patients were categorized as red (4.4%), orange (25.2%), yellow (38.7%), and green (31.7%). Abnormal vital signs were present at admission in 25% of the patients, most often temperature (10.5%), saturation of peripheral oxygen (9.2%), Glasgow Coma Score (6.6%), and respiratory rate (4.8%). A venous acid-base status was obtained in 43% of all patients. The majority (78%) had a pH within the normal range (7.35–7.45), 15% had acidosis (pH < 7.35) and 7% had alkalosis (pH > 7.45). Median length of stay was 2 days (range 1–123). The proportion of patients admitted to Intensive Care Unit was 1.6% (95% CI 1.2-2.0), 1.8% (95% CI 1.5–2.2) died within 7 days, and 4.2% (95% CI 3.7–4.7) died within 28 days after admission.	Despite challenges of data registration, we succeeded in creating a database of adequate size and data quality. Future studies will focus on the association between patient status at admission and patient outcome, e.g., admission to Intensive Care Unit or in-hospital mortality.
American College of Emergency Physicians Clinical Emergency Data Registry (CEDR)	Venkatesh, 2021 [24]	To develop a volume-adjusted ED throughput quality measure to balance variation at the ED group level.	We found marked differences in the classification of ED throughput performance between scoring approaches. The weighted standardized score (z score) approach resulted in the least skewed and most uniform distribution across the majority of ED types, with a kurtosis of 12.91 for taxpayer identification numbers composed of 1 ED, 2.58 for those with multiple EDs without any supercenter, and 3.56 for those with multiple EDs with at least 1 supercenter, all lower than comparable scoring methods. The plurality and simple average scoring approaches appeared to disproportionally penalize ED groups that staff a single ED or multiple large-volume EDs.	Application of a weighted standardized (z score) approach to ED throughput measurement resulted in a more balanced variation between different ED group types and reduced distortions in the length-of-stay measurement among ED groups staffing high-volume EDs. This approach may be a more accurate and acceptable method of profiling ED group throughput pay-for-performance programs.
Centre des Urgences de Yaoundé (CURY)	Jeong, 2022 [54]	This paper describes the methods of CURY patient data collection and the characteristics of the patients visited CURY from January 2016 to June 2018.	During the study period, a total of 18,875 patients’ data were collected (44.5% women, median age of 36). Of the total patients, 2.4% had chest pain, 2.7% had stroke, 1.9% had sepsis/septic shock, and 1.6% had multiple trauma. About 6.0% of patients received operation and majority of patients were discharged either normally (48.2%) or with continuity of care (26.3%). About 5.0% of patients were transferred to other hospitals, and 5.2% of patients were dead.	This study serves to broaden understanding of the emergency patients in Yaoundé, Cameroon. The hospital patient database for emergency patients can be further used as a basis for providing improved quality of medical care and effective communication tools among the medical staffs.
Danish Database for Acute and Emergency Hospital Contacts (DDAEHC)	Lassen, 2016 [48]	The aim of the Danish database for acute and emergency hospital contacts (DDAEHC) is to monitor the quality of care for all unplanned hospital contacts in Denmark (acute and emergency contacts).	The DDAEHC also includes age, sex, Charlson Comorbidity Index conditions, civil status, residency, and discharge diagnoses. The DDAEHC expects to include 1.7 million acute and emergency contacts per year.	The DDAEHC is a new database established by the Danish Regions including all acute and emergency hospital contacts in Denmark. The database includes specific outcome and process health care quality indicators as well as demographic and other basic information with the purpose of being used for enhancement of quality of acute care.
Swedish Emergency Registry (SVAR)	Ekelund, 2011 [47]	To assess the feasibility of collecting selected quality of care data from six different Swedish EDs using automated data capture as a basis for a national quality of care registry, and to present some first results regarding throughput times and patient presentation times.	All EDs provided throughput times and patient presentation data without significant problems. In all EDs, Monday was the busiest day, and the fewest patients presented on Saturday. All EDs had a large increase in patient inflow before noon with a slow decline over the rest of the 24 h, and this peak and decline was especially pronounced in elderly patients. The average LOS was 4h of which 2h was spent waiting for the first physician. These throughput times showed a considerable diurnal variation in all EDs, with the longest times occurring 6-7am and in the late afternoon.	These results demonstrate the feasibility of collecting benchmarking data on quality of care targets within Swedish EM, and form the basis for ANSWER, A National SWedish Emergency Registry.
The Registry for Emergency Care	O’Reilly, 2020 [39]	The first objective of the REC Project is to determine the impact of patient isolation and IPC processes on ED length of stay for adult patients.	Clinical tools will be generated to inform emergency care, both during and beyond the COVID-19 pandemic.	The REC Project will support ED clinicians in the emergency care of all patients.

**Table 4:** General characteristics of ED clinical quality registries.

Registry		Date Range	Country/ies	Number of EDs ^3^	Condition or Population	Funding	Associated Studies ^2^
Acute Admission Database	Barford, 2012 [74]	22 September 2009 to 28 Feburary 2010	Denmark	1	General	Hillerød Hospital research grant.	0
American College of Emergency Physicians Clinical Emergency Data Registry (CEDR)	Venkatesh, 2021 [24]	2017 ^1, 5^	USA	889	General	American College of Emergency Physicians	0
Centre des Urgences de Yaoundé (CURY) ^4^	Jeong, 2022 [54]	January 2016 to June 2018 ^1^	Africa	1	General	Korea International Cooperation Agency	0
Danish Database for Acute and Emergency Hospital Contacts (DDAEHC)	Lassen, 2016 [48]	Not specified ^5^	Denmark	26	General	Danish Regions.	0
Swedish Emergency Registry (SVAR)	Ekelund, 2011 [47]	1 January 2009 to 30 June 2009 ^1, 5^	Sweden	6	General	Region Skåne, the Stockholm County Council and the Swedish Association of Local Authorities and Regions.	1
The Registry for Emergency Care (REC)	O’Reilly, 2020 [39]	Not specified	Australia	1	General	No funding acknowledged.	1
Cleveland Clinic Emergency Airway Registry ^4^	Phelan, 2010 [34]	1 July 2005 to 31 March 2007	USA	1	Airway	No funding acknowledged.	0
Australia and New Zealand Emergency Department Airway Registry (ANZEDAR)	Fogg, 2016 [60]	1 April 2010 to 30 March 2014 ^5^	Australia	1	Airway	Emergency Care Institute research funding scheme.	3
Defense Registry for Emergency Airway Management (DREAM)	Mendez, 2021 [43]	January 2020 to July 2020 ^1^	USA	1	Airway	No funding acknowledged.	0
Korean Emergency Airway Management Registry (KEAMR)	Choi, 2012 [63]	March 2006 to December 2010 ^1^	Korea	13	Airway	No funding acknowledged.	2
National Emergency Airway Registry (NEAR)	Brown, 2015 [49]	1 July 2002 to 31 December 2012 ^5^	USA, Australia, Canada	13	Airway	No funding acknowledged.	19
South African Emergency Department Airway Registry ^4^	Hart, 2020 [56]	1 September 2015 to 31 October 2016 ^1^	South Africa	1	Airway	No funding acknowledged.	0
Children’s Injury Database (CID)	McCain, 2023 [44]	2021 ^1^	USA	1	Pediatric	No funding acknowledged.	0
Nicaragua Pediatric Emergency Registry ^4^	Bressan, 2021 [71]	1 January 2017 to 31 December 2017 ^1^	Nicaragua	7	Pediatric	Regione Lombardia and the Associazione il Bambino Nefropatico	0
The Pediatric Emergency Care Applied Research Network Registry (PECARN)	Davies, 2018 [35]	January 2012 to June 2016 ^1, 5^	USA	7	Pediatric	Agency for Healthcare Research and Quality ^6^.	0
Emergency Medicine Pulmonary Embolism in the Real World Registry (EMPORER)	Pollack, 2011 [33]	1 January 2005 to 29 December 2008	USA	22	Acute pulmonary embolism	GlaxoSmithKline.	1
Risk Profile of Patients VTED Attended in Spanish Emergency Departments Registry (ESPHERIA)	Jimenez, 2017 [53]	13 October 2014 to 14 December 2014 ^1^	Spain	53	Venous thromboembolism	Bayer Hispania.	1
Emergency Atrial Fibrillation Registry of the Catalan Institute of Health (URGFAICS)	Jacob, 2019 [55]	September 2016 to February 2017 ^1^	Spain	5	Atrial fibrillation	No funding acknowledged.	1
Epidemiology of Acute Heart Failure in Emergency Departments Registry (EAHFE)	Llorens, 2015 [45]	15 March to 15 May 2007; 1 June to 30 June 2009; 7 November 2011 to 7 January 2012	Spain	29	Heart failure	Partially funded by the Institute of Health.	2
Acute Epileptic Seizures in the Emergency Department Registry (ACESUR)	Alonso, 2019 [58]	1 February 2017 to 31 October 2017	Spain	18	Acute epileptic seizures	No funding acknowledged.	0
Ain Shams University Hospital Trauma Registry ^4^	Khalil, 2021 [51]	January 2017 to December 2017	Egypt	1	Trauma	Fogarty Institute in USA.	0
Auckland City Hospital Emergency Department Overdose Database	Theron, 2007 [27]	2002 to 2004 ^1^	New Zealand	1	Overdose	No funding acknowledged.	0
Emergency Medicine Events Register (EMER)	Hansen, 2016 [57]	December 2012 to February 2015 ^1^	Australia, New Zealand	21	Safety incidents	Australasian College for Emergency Medicine	0
Procedural Sedation in the Community Emergency Department Registry (ProSCED)	Sacchetti, 2007 [30]	1 January 2003 to 4 March 2006 ^1^	USA	14	Procedural sedation	No funding acknowledged.	1
Singapore Head Injury Database ^4^	Chong, 2015 [62]	January 2006 to June 2014 ^1^	Singapore	1	Pediatric head injury	Pediatrics Academic Clinical Program, Singapore.	0
The Sepsis Registry ^4^	Williams, 2011 [23]	Not specified	Australia	1	Sepsis	Queensland Emergency Medicine Research Foundation.	0
VNICat (NIVCat in English)	Jacob, 2019 [55]	February 2015 to March 2015 ^1^	Spain	8	Non-invasive mechanical ventilation	No funding acknowledged.	1

^1^ No specific day and/or month described in the methodology. ^2^ Found with the original search strategy. ^3^ Based on the most recent publication identified in the search strategy. ^4^ A name for the registry not mentioned in the manuscript. ^5^ Registry ongoing. ^6^ The PECARN infrastructure was supported by the Health Resources and Services Administration (HRSA), the Maternal and Child Health Bureau (MCHB), and the Emergency Medical Services for Children (EMSC) Network Development Demonstration Program.

## Data Availability

The original contributions presented in the study are included in the publication/Supplementary Material; further inquiries can be directed to the corresponding author/s.

## Supplementary Materials

The following supporting information can be downloaded at: https://www.mdpi.com/article/10.3390/healthcare13091022/s1, Table S1: Preferred Reporting Items for Systematic reviews and Meta-Analyses extension for Scoping Reviews (PRISMA-ScR) Checklist; Table S2: Aims, results and conclusion of primary ED registry publications specific for a condition or population; Table S3: Aims, results, and conclusion of studies secondary publications (both general scope and specific for a condition or population).

## Appendix A. Search Strategy

The following electronic databases were searched: National Library of Medicine via PubMed, Embase, and Web of Science as a minimum requirement to guarantee adequate and efficient coverage (Table A1) [22]. The search period included date of database inception to April 2024. For all databases, only publications in English and where full text was available were included.

For the National Library of Medicine, the PubMed Central search field query entered was: (((registry[Title]) OR register[Title]) OR database[Title]) AND emergency[Title]. For embase, we used emtree terms; ‘registry’ or ‘register’ or ‘database’ combined with the MeSH terms referring to ‘emergency ward’ or ‘emergency medicine’ or ‘emergency care’. For the Web of Science we searched in “All Databases”, “All” collections, document type ‘article’, language English. We used the search terms: (((SO = (emergency)) AND SO = (registry)) OR SO = (register)) OR SO = (database).

Medical Subject Headings (MeSH), ‘registries’ or ‘database’ AND ‘emergency medicine’ or ‘emergency room visits’ or ‘emergency medical service’ or ‘evidence based emergency medicine’.

**Table A1:** Detailed search strategy for PubMed, Embase, and Web of Science.

	Search Query
All databases	Full text available; English language
PubMed	((Emergency [Title]) AND (Registry [Title] OR Register[Title] OR Database[Title]))
Embase	((Emergency.ti.) AND (Registry.ti. OR Register.ti. OR Database.ti.))
Web of Science	(TI=(Emergency) AND (TI = (Registry) OR TI = (Register) OR TI = (Database)))
