# Supplementary material for: Emergency Department Clinical Quality Registries: A Scoping Review
Source: Healthcare (Basel). 2025 Apr 29;13(9):1022. doi: 10.3390/healthcare13091022 (PMC12071968; doi:10.3390/healthcare13091022)
Supplement: Supplementary file 1 [file healthcare-13-01022-s001.zip › Scoping Review ED CQRs Supp 2.pdf]

Supplementary 2. Aims, results and conclusion of Primary ED registry publications specific for a condition or population

Registry	Aim	Results	Conclusion
Cleveland Clinic Emergency Airway Registry<br>*Phelan, 2010 [34]*	The aim of this study was to determine if an airway registry (ie, an airway data collection sheet that is filled out at the point of care and the information then entered into a database) can be used as a tool to survey and evaluate EM airway management practices in a busy urban ED and if the collected data can eventually be used to benchmark or compare with a standard.	An audit form was developed and implemented to collect data on intubations. During the study period, 224 patients required invasive airway control. Of all airways managed by emergency medicine residents, the intubation success rate was 99% (200/203; 95% confidence interval [CI] = 96%-100%), with 3% of those (6/203; 95% CI = 1%-6%) requiring more than 3 attempts; 3 patients (1%; 95% CI = 0%-4%) could not be intubated and required a surgical airway.	Use of an airway registry based on the NEAR registry as a benchmark of rates and types of successful intubation allows comparison of airway practices.
Australia and New Zealand Emergency Department Airway Registry (ANZEDAR)<br>*Fogg, 2016 [60]*	To investigate whether a bundle of changes made to the practice of endotracheal intubation in our ED was associated with an improvement in first pass success rate and a reduction in the incidence of complications.	The data on 360 patients who were intubated during an 18-month period following the introduction of these changes were compared with our previously published observational data. Success on first attempt at intubation improved 83.4% to 93.9% ($P < 0.0001$). The proportion of patients with one or more complication fell from 29.0% to 19.4% ($P < 0.042$). Esophageal intubation fell from 4.0% to 0.3% ($P < 0.001$), and there was a non-significant reduction in the rate of desaturation, from 15.6% to 10.9% ($P<0.07$).	We have shown that, through the introduction of a bundle of changes that spans the domains of staff training, equipment and practice standardization, we have made significant improvements in the safety of patients undergoing endotracheal intubation in our ED.
Defense Registry for Emergency Airway Management (DREAM)<br>*Mendez, 2021 [43]*	We describe the pilot data collected as part of the development of the DREAM at BAMC- the Department of Defense's (DoD) only level 1 trauma center.	The study period comprised January through July 2020. During the study period emergency physicians (EP) performed a total of 74 intubations. Reasons for intubation were related to trauma for 47 patients (64%) and medical conditions for 26 patients (36%). The median age was 51 (interquartile range 30-72) and most were male 48 (65.7%). Difficult airway characteristics encountered included blood in the airway (26%), facial trauma (23%), and airway obstruction (1%). Most intubations utilized video laryngoscopy, and the most frequently used airway devices were Macintosh-shaped (45%) and hyperangulated-shaped (41%). Overall, first-pass success rate was 93% (69) with majority of intubations performed by second-year emergency residents (61%) followed by first-year residents (28%).	Most DREAM intubations were related to traumatic injuries. The most frequently encountered difficult airway characteristics were blood in airway and facial trauma. Most intubations were conducted using video laryngoscopy with a high first-pass success rate similar to other published studies. Expansion of the registry to other military emergency departments would enable a data-driven solution for development of individual critical task lists.
Korean Emergency Airway Management Registry (KEAMR)<br>*Choi, 2012 [63]*	To investigate the factors associated with successful first-attempt pediatric endotracheal intubation (ETI) in the emergency department from multicenter emergency airway registry data.	A total of 430 ETIs were performed on 281 children seen in the ED. The overall first-pass success (FPS) rate was 67.6%, but emergency medicine (EM) physicians showed a significantly greater success rate of 74.4%. In the logistic regression analysis, the intubator's specialty was the only independent predictive factor for pediatric FPS. In the subgroup analysis, the EM physicians used the rapid sequence intubation (RSI) method and Macintosh laryngoscope more frequently than physicians of other specialties. ETI-related adverse events occurred in 21 (7.2%) out of the 281 cases. The most common adverse event in the FPS group was mainstem bronchus intubation, and vomiting was the most common event in the	The intubator's specialty was the major factor associated with FPS in emergency department pediatric ETI. The overall ETI FPS rate among pediatric patients was 67.6%, but the EM physicians had a FPS rate of 74.4%.

		non-FPS group. The incidence of adverse events was lower in the FPS group than in the non-FPS group, but this difference was not statistically significant.	
National Emergency Airway Registry (NEAR) *Brown, 2015 [49]*	To characterize ED intubation practices spanning a decade of experience to analyze performance attributes and identify evolving trends.	Of 18 participating centers, 5 were excluded for failing to meet compliance standards. From the remaining 13 centers, we report data on 17,583 emergency intubations of patients aged 15 years or older from 2002 to 2012. Indications were medical in 65% of patients and trauma in 31%. Rapid sequence intubation was the first method attempted in 85% of encounters. Emergency physicians managed 95% of intubations and most (79%) were physician trainees. Direct laryngoscopy was used in 84% of first attempts. Video laryngoscopy use increased from less than 1% in the first 3 years to 27% in the last 3 years (risk difference 27%; 95% CI 25% to 28%; mean odds ratio increase per year [ie, slope] 1.7; 95% CI 1.6 to 1.8). Etomidate was used in 91% and succinylcholine in 75% of rapid sequence intubations. Among rapid sequence intubations, rocuronium use increased from 8.2% in the first 3 years to 42% in the last 3 years (mean odds ratio increase per year 1.3; 95% CI 1.3 to 1.3). The first-attempt intubation success rate was 83% (95% CI 83% to 84%) and was higher in the last 3 years than in the first 3 (86% versus 80%; risk difference 6.2%; 95% CI 4.2% to 7.8%). The airway was successfully secured in 99.4% of encounters (95% CI 99.3% to 99.6%).	In the EDs we studied, emergency intubation has a high and increasing success rate. Both drug and device selection evolved significantly during the study period.
South African Emergency Department Airway Registry *Hart, 2020 [56]*	To analyze first-pass as well as direct laryngoscopy (DL) and video laryngoscopy (VL) success rates; to assess the adequacy of pre-oxygenation; and to compare adverse event (AE) rates and factors affecting patient deterioration during airway management in an South African (SA) ED with international data.	A total of 321 intubations were included. The majority of the patients (71.6%) had non-traumatic indications for intubation. The overall first-pass intubation success (FPS) rate for doctors was 81.8%. Although this rate is lower than the mean rate suggested in an international meta-analysis (84.1%), it is within the 95% confidence interval (80.1 - 87.4%). Overall FPS rates showed no difference between video laryngoscopy (81.7%) compared with direct laryngoscopy (73.3%) (p-value 0.079), although better glottic views were obtained with video laryngoscopy (80.5% were Cormack-Lehane grade 1). Analysis of pre-oxygenation methods found that although sicker patients had received more aggressive pre-oxygenation, e.g. with non-invasive or bag-mask ventilation techniques, they still desaturated more often (35.8% and 62.5%, respectively) than less sick patients who received simple non-rebreather facemask pre-oxygenation (4.5%).	This analysis of the first airway registry from an SA ED highlights that airway management in an LMIC can be performed on par with accepted international standards.
Children's Injury Database (CID) *McCain, 2023 [44]*	In an effort to more clearly define the burden injuries cause in our catchment area, our research team designed the Children's Injury Database (CID). This database is an injury surveillance system that collects information regarding injured children aged 0–16 years who are treated (and then are discharged home, are transferred to another facility, or are admitted to the hospital) in an urban free standing tertiary pediatric ED with an	During 2021, a total of 15,168 injury visits were analyzed representing 22% of total ED visits (68,834). A total of 2053 injury visits (13.5%) resulted in hospital admission. The 10 leading injury types included: falls, poisonings, motor vehicle collision (MVC), assault, dog bite, burns, sports, pedestrian, bicycle, and all-terrain vehicle (ATV). Admission rates varied by age group with children ages 13 years and older having the highest rate of admission (18.4%). The median length of stay (LOS) for all injured children requiring admission was 2 days while the median LOS for preschoolers was 1 day, the median LOS for school-age children was 2 days, and the median LOS for teenagers was 3 days. While MVCs were the most common cause of vehicle-related injuries, ATV-related injuries had the highest rate of admission (51%).	In this study, teenagers had significantly higher admission rates, lengths of stay, and hospital charges. Black and Hispanic children were under-represented in the number of visits for injuries compared to all ED visits. Further research should focus on disparities in injury-related visits based on race as well as gender. CID has demonstrated that injury surveillance systems can assist with reporting new injury patterns while also acting as a stimulus for new research ideas, planning interventions targeting

	annual patient volume of 74,000 patients per year.		the most at-risk populations, and evaluating the effectiveness of injury prevention interventions.
Nicaragua Pediatric Emergency Registry<br>*Bressan, 2021 [71]*	To describe the characteristics and outcomes of the severe spectrum of pediatric emergency visits using a multi-site registry as developed as part of an international project over a one-year period.	Overall, 3521 visits of patients <15 years of age, of whom two-thirds <5 years, met predefined inclusion criteria of urgent-emergency visits. Respiratory (1619/3498; 46%), gastrointestinal (407/3498; 12%) and neurological (368/3498; 11%) complaints were the most common symptoms. Malnutrition was reported in 18% (610/3448) of presentations. Mortality was 7% (233/3521); 52% (120/233) of deaths occurred in the <1-year subgroup; 32% (71/3521) of deaths occurred within the first 24 hours of presentation. The most common immediate causes of death were septic shock (99/233; 43%), respiratory failure (58/233; 25%) and raised intracranial pressure (24/233; 10%).	The mortality rate of urgent-emergency pediatric visits in Nicaragua is high, with younger children being most at risk and the majority of deaths being eventually caused by septic shock or respiratory failure. Our data provide useful information for the development of a Pediatric Emergency Care network to help direct training efforts, resources and logistic/organizational interventions to improve children's health in an emergency setting in Nicaragua.
The Pediatric Emergency Care Applied Research Network Registry (PECARN)<br>*Sara, 2018 [80]*	Describe the creation of the PECARN Registry, which is part of a larger project funded by the Agency for Healthcare Research and Quality (AHRQ) to use EHR-derived data to provide benchmarked, stakeholder-endorsed, quality metrics as well as audit and feedback to emergency providers on their care derived from these metrics.	The Pediatric Emergency Care Applied Research Network (PECARN) Registry, representing four hospital systems and seven EDs, demonstrates that ED data from disparate health systems and EHR vendors can be harmonized for use in a single registry with a common data model. The current PECARN Registry represents data from 2,019,461 pediatric ED visits, 894,503 distinct patients, more than 12.5 million narrative reports, and 12,469,754 laboratory tests and continues to accrue data monthly.	The Registry is a robust harmonized clinical registry that includes data from diverse patients, sites, and EHR vendors derived via data extraction, deidentification, and secure submission to a central data coordinating center. The data provided may be used for benchmarking, clinical quality improvement, and comparative effectiveness research.
Emergency Medicine Pulmonary Embolism in the Real World Registry (EMPEROR)<br>*Pollack, 2011 [33]*	To establish more definitively the presentation symptoms and signs of Pulmonary Embolism (PE) presenting in the ED setting; characterize treatment used by U.S. emergency physicians; and measure use of risk stratification methods, frequencies of empiric anticoagulation use, and rates of major hemorrhage and mortality among patients who present to the ED.	A total of 1,880 patients with confirmed acute PE were enrolled from 22 U.S. EDs. diagnosis of PE was based upon positive results of computerized tomographic pulmonary angiogram in most cases (n = 1,654 [88%]). Patients represented both sexes equally, and racial and ethnic composition paralleled the overall U.S. ED population. Most (79%) patients with PE were employed, and one-third were older than age 65 years. The mortality rate directly attributed to PE was 20 in 1,880 (1%; 95% confidence interval [CI]: 0% to 1.6%). Mortality from hemorrhage was 0.2%, and the all-cause 30-day mortality rate was 5.4% (95% CI: 4.4% to 6.6%). Only 3 of 20 patients with major PE that ultimately proved fatal had systemic anticoagulation initiated before diagnostic confirmation, and another 3 of these 20 received a fibrinolytic agent.	Patients diagnosed with acute PE in U.S. EDs have high functional status, and their mortality rate is low. These registry data suggest that appropriate initial medical management of ED patients with severe PE with anticoagulation is poorly standardized and indicate a need for research to determine the appropriate threshold for empiric treatment when PE is suspected before diagnostic confirmation.
Risk Profile of Patients VTED Attended in Spanish Emergency Departments Registry (ESPHERIA)<br>*Jimenez, 2017 [53]*	To determine the clinical profile of and the diagnostic and therapeutic approach to patients diagnosed with venous thrombo-embolism (VTE), deep vein thrombosis (DVT) and PE in the ED.	From 549,840 ED visits made over a mean period of 40 days, 905 patients were diagnosed with VTE (incidence 1.6 diagnoses per 1000 visits). The final analysis included 801 patients, of whom 49.8% had pulmonary embolism. The most frequent risk factors for VTE were age (≥70 years), obesity, and new immobility. Clinical probability, prognosis, and bleeding risk scales were recorded in only 7.6%, 7.5%, and 1% of cases, respectively. D-dimer was determined in 87.2% of patients with a high clinical probability of VTE, and treatment was initiated before confirmation in only 35.9% of these patients. In patients with PE, 31.3% had a low	VTE had a substantial impact on Spanish EDs. The clinical presentation and risk profile for the development of VTE in patients diagnosed in the EDs was similar to that recorded in previous studies. During follow-up, bleeding (overall) was more frequent than recurrences. Adherence to clinical practice guidelines could improve significantly.

		risk of VTE. Overall, 98.7% of patients with pulmonary embolism and 50.2% of patients with DVT were admitted. During follow-up, total bleeding was more frequent than recurrences: the rates of any bleeding event were 4.4%, 3.9%, 5.3%, and 3.5% at admission and at 30 and 90, and 180 days, respectively; the rates of VTE recurrence were 2.3%, 1.3%, 1.7%, and 0.6%, respectively. Mortality rates were 3.4%, 3.1%, 4.1%, and 2.6% during hospitalization and at 30, 90, and 180 days, respectively.	
Emergency Atrial Fibrillation Registry of the Catalan Institute of Health (URGFAICS) *Jacob, 2019 [55]*	To investigate the characteristics of patients visiting the ED for an episode of atrial fibrillation (AF) depending on whether AF is de novo or previously diagnosed and to quantify emergency re-visits related to AF at 30 days and its associated factors.	Of the 1199 patients included in the registry, 225 patients (18.8%) were treated with amiodarone while 974 (81.2%) were not. We performed a univariate study depending on amiodarone administration followed by propensity score calculation according to the 14 statistically different features found previously and six significant variables, obtaining 150 patients (75 for each group) suitable for the analysis. The length of ED stay was analyzed using box plot, with a $P < 0.001$ in the crude analysis and $P = 0.012$ after propensity score matching and using survival curves for the analysis of prolonged ED stay, with a log rank <0.001 in the crude analysis and log rank 0.021 after the propensity score-matched analysis.	Amiodarone is associated with longer length of ED stay until discharge independently of the baseline characteristics of the patients.
Epidemiology of Acute Heart Failure in Emergency Departments Registry (EAHFE) *Llorens, 2015 [45]*	1) to describe the clinical characteristics, laboratory findings, treatment and outcome of acute heart failure (AHF) patients seen in Spanish EDs; 2) to detect changes in the management of these patients in such EDs over time; and 3) compare the results of this registry with those of other published AHF registries.	A total of 5845 patients were included (2007, 948; 2009, 1483; 2011, 3414). The mean age was 79 years and 56% were women. The AHF episode registered was the first experienced by 34.6% of the patients. Comorbidity was high: 82% had hypertension, 42.3% had diabetes mellitus, and 47.7% had AF. Severe or total functional dependence was observed in 21.9%, and 57.3% had systolic dysfunction (left ventricular ejection fraction, 38.3%). The main treatments administered consisted in diuretics (96.8%), endovenous nitroglycerine (20.7%), noninvasive ventilation (6.4%), and inotropic agents or vasopressors (3.6%). The glomerular filtration rate was low in 57%. Troponin and natriuretic peptide levels were measured in the EDs in 49.1% and 42.4% of the cases, respectively. Patients presented as normotensive in 66.4% of the cases, hypertensive in 23.5%, and hypotensive in 4.6% (0.7% in shock); 76.1% were admitted (1.9% to the ICU). The median hospital stay was 7 days and 23.9% were discharged from the ED. In-hospital mortality was 7.6%; 30-day mortality was 9.4% and 1-year mortality 29.5%. Orders for troponin and natriuretic peptide determinations increased over the 3 study periods, and the intravenous infusion of diuretics and inotropic agents and vasoconstrictors decreased ($P < 0.001$, all comparisons). Revisits within 30 days also decreased ($P = 0.004$). No changes were observed in in-hospital or 30-day mortality rates between 2007 and 2011. We reviewed 14 previously published registry reports (8 compiled prospectively); only 2 of the registries included ED patients.	The EAHFE registry describes the characteristics of AHF in a cohort that resembles the universe of our patients with AHF. Significant changes were observed over time in some aspects of AHF management. Revisits decreased, but mortality rates remained unchanged. Only 2 other previously analyzed registries included patients with AHF treated in hospital EDs.
Acute Epileptic Seizures in the Emergency Department Registry (ACESUR) *Alonso, 2019 [58]*	To describe the characteristics of care received by patients who come to the emergency department with a first epileptic seizure versus a recurrent seizure in a patient with diagnosed epilepsy.	A total of 664 patients attended by 18 Spanish emergency departments were entered into the ACESUR registry. Two hundred twenty-nine (34.5%) were first seizures and 435 (65.5%) were recurrences. Patients who were attended for first seizures were older, consulted for a wider variety of reasons, and were transported in ambulances ($P<.001$, all comparisons). Care received differed between patients with first seizures versus recurrent seizures. Specific complementary testing was	The clinical characteristics of adults attended for a first epileptic seizure differ from those of patients with diagnosed epilepsy who were attended for recurrent seizures in Spain. The care received also differs.

		more likely in patients with first seizures (adjusted odds ratio [aOR], 13.94; 95% CI, 29-26.7; P<.001), and they were more often hospitalized or stayed longer in the emergency department, (aOR, 1.69; 95% CI, 1.11-2.58; P=.015). Pharmacologic treatment did not differ between the groups, either in the acute phase or for prevention (aOR, 1.40; 95% CI, 0.94-2.09; P=.096). Antiepileptic drugs were given to 100 patients (43.7%) after a first seizure and were restarted or changed in 142 patients with recurrent seizure (32.6%).	
Ain Shams University Hospital Trauma Registry *Khalil, 2021 [51]*	The present study aimed to introduce a basic trauma registry for the emergency department of Ain Shams University Hospital, one of the tertiary care hospitals involved in trauma management within Cairo.	The collected data were statistically analyzed, after which the epidemiological distribution of the trauma patients was described. Accordingly, our results showed that among the included patients, 65% were men, whereas most were single, illiterate, and unemployed. Thereafter, statistics regarding trauma circumstances were generated.	In conclusion, the present study found that hospital trauma registries can be established, are essential for improving the quality of health care services, and should be used ubiquitously throughout the country's hospitals to establish a national registry.
Auckland City Hospital Emergency Department Overdose Database *Theron, 2017 [27]*	To examine the impact of 'party pills' (PP; herbal highs) on the Auckland City Hospital Emergency Department Overdose Database 2002-2004, and to present figures for five other substances in that database.	In 2002, 1 patient presented with PP ingestion; 4 presented in 2003 and 21 in 2004 respectively (p<0.001). Of these 21 patients in 2004, 5 had allegedly ingested PP only and none required medical admission. PP only contributed to 1.58% of the overdose database for 2004.	Party pills' appeared to have a minor impact on the overdose database at Auckland City Hospital between 2002 and 2004. There was a significant decrease in GHB presentations from 2003 to 2004 (p<0.001), but no significant fall in stimulant overdose presentations.
Emergency Medicine Events Register (EMER) *Hansen, 2016 [57]*	To conduct an analysis on the first 150 incidents entered into the Emergency Medicine Events Register (EMER).	Over the first 26 months, 150 incidents were reported into EMER. The most common categories reported, in order, were diagnostic error, procedural complication and investigation errors. Most incidents contained more than one category of error. The most common stage of the patient's journey in which an incident was detected was after discharge from the ED.	A focus on correct diagnosis, procedure performance and investigation interpretation may reduce errors in the ED. The ability to learn from incidents and make system changes to enhance patient safety in healthcare organizations is an inherent part of providing a proactive, quality culture.
Procedural Sedation in the Community Emergency Department Registry (ProSCED) *Sacchetti, 2007 [30]*	The Procedural Sedation in the Community Emergency Department (ProSCED) registry was developed to examine specifically the PSA practices of community EPs. In addition to profiling community PSA activities, the ProSCED registry was designed to determine if community EPs can deliver safe and effective procedural sedation.	A total of 1,028 procedural sedations were performed on 980 patients at 14 study sites. The most common specified procedures performed included shoulder relocation (392), hip relocation (102), elbow relocation (70), upper extremity fracture care (69), lower extremity fracture care (66), and facial laceration repair (67). Complications of any description occurred in 42 cases (4.1%), with no patient's disposition changed secondary to a complication. Patients' ages ranged from 1 month to 95 years, with a median age of 31 years. Of procedures attempted, 982 (95.5%) were successfully completed, 21 cases (2.0%) were adequately sedated but unable to have their procedure completed, and 21 cases (2.0%) were believed to be inadequately sedated. Medication use included midazolam in 423 cases (41.1%), propofol in 253 (24.6%), fentanyl in 253 (24.6%), etomidate in 241 (23.4%), and ketamine in 145 (14.1%), as well as several others. Cases using either ketamine or propofol exhibited the fewest complications, while those using fentanyl, hydromorphone, or midazolam demonstrated the highest complication rates.	Community emergency physicians deliver safe and effective PSA over a wide variety of ages and procedures while using a broad selection of agents.

Singapore Head Injury Database *Chong, 2015 [62]*	To derive clinical predictors for moderate to severe head injury that would guide physicians facing a head injured child.	There were 39 cases and 1173 controls. In the prospective database, our event rate was 0.5% and our computed tomography (CT) rate was 1%. Among those with moderate to severe head injury, they were more likely to be involved in road traffic accidents, have a history of difficult arousal, confusion or disorientation and a history of seizure. On physical examination, cases were more likely to have the presence of altered mental status, base of skull fracture, scalp hematoma and anisocoria. On multivariable analysis, the following 4 predictors remained statistically significant: Involvement in road traffic accident ($p < 0.001$), difficult arousal ($p < 0.001$), vomiting ($p = 0.003$) and signs of base of skull fracture ($p < 0.001$). Using these 4 variables, the Area under Curve was 0.97 {Sensitivity 92.3% (79.1-98.4%), Specificity 93.0% (91.4-94.4%), positive predictive value 30.5% (22-40%), negative predictive value 99.7% (99.2-99.9%)}.	Involvement in road traffic accident, difficult arousal, base of skull fracture and vomiting are independent predictors for moderate to severe head injury in our pediatric population.
The Sepsis Registry *Williams, 2011 [23]*	The aim of this registry is to systematically collect quality observational clinical and microbiological data regarding emergency patients admitted with infection, in order to explore in detail the microbiological profile of these patients, and to provide the foundation for a significant program of prospective observational studies and further clinical research.	This database will provide substantial insights into the characteristics, microbiological profile, and outcomes of emergency patients admitted with infections. It will become the nidus for a program of research into compliance with evidence-based guidelines, optimization of empiric antimicrobial regimens, validation of clinical decision rules and identification of outcome determinants. The detailed observational data obtained will provide a solid baseline to inform the design of further controlled trials planned to optimize treatment and outcomes for emergency patients admitted with infections.	No conclusion section
VNICat (NIVCat in English) *Jacob, 2019 [55]*	The objective of the NIVCat registry (VNICat in Spanish) was to describe the characteristics of the patients in whom non-invasive ventilation (NIV) is performed in prehospital and hospital emergencies and to investigate if there is any clinical scenario that is related to worse survival results.	We studied 184 acute episodes requiring NIV, in the prehospital setting in 25 cases (13.6%) and in the hospital in 159 (86.4%). The most common scenario was acute heart failure (AHF) (38.0%). The second most common was chronic obstructive pulmonary disease (COPD) (34.2%). In most cases, NIV was discontinued in the emergency department. Mortality was 7.5% during prehospital care and 21.4% in the hospital. Hospital mortality was associated with limiting the use of life support. We detected no significant differences in mortality between the groups of patients with AHF vs COPD.	The use of NIV in prehospital and hospital emergency care follows current evidence-based recommendations and is required more often for AHF than for exacerbated COPD. Hospital mortality is high in this context and is associated with frequent limiting of life support.
