# Supplementary material for: Emergency Department Clinical Quality Registries: A Scoping Review
Source: Healthcare (Basel). 2025 Apr 29;13(9):1022. doi: 10.3390/healthcare13091022 (PMC12071968; doi:10.3390/healthcare13091022)
Supplement: Supplementary file 1 [file healthcare-13-01022-s001.zip › Scoping Review ED CQRs Supp 3.pdf]

Supplementary 3. Aims, results and conclusion of secondary publications (both general scope and specific for a condition or population)

Secondary Study	Aims	Results	Conclusion
**Swedish Emergency Registry (SVAR)** *Ekelund, 2011 [47]*			
Emergency department crowding and mortality in 14 Swedish emergency departments, a cohort study leveraging the Swedish Emergency Registry (SVAR).	To investigate the association between ED crowding and all-cause mortality within 7 and 30 days from the ED visit, and the potential differences between three counties in Sweden.	2,440,392 visits from 1,142,631 unique patients were analyzed. A significant association was found between crowding and 7-day mortality but not with 30-day mortality. Subgroup analysis also yielded mixed results with a clear association in only one of the three counties. The estimated HR (95% CI) for 30-day mortality for admitted patients in this county was 1.06 (1.01-1.12) in the moderate crowding category, and 1.11 (1.01-1.22) in the high category.	The association between crowding and mortality may not be universal. Factors that influence the association between crowding and mortality at different EDs are still unknown but a high hospital bed occupancy, impacting admitted patients may play a role.
**The Registry for Emergency Care (REC)** *O'Reilly, 2020* [39]			
Impact of patient isolation on emergency department length of stay: A retrospective cohort study using the Registry for Emergency Care.	To determine if isolation is associated with an increased length of stay in the ED.	There were 447 patients who met inclusion criteria, of which 123 (28%) were managed in isolation. The median (interquartile range) ED LOS was 259 (210-377) min for the isolation group and 204 (126-297) min for the non-isolation group, a difference in median ED LOS of 55 min (P < 0.001). Isolation was independently associated with a 23% increase in ED LOS (P = 0.002) and doubled the odds of an ED stay of more than 4 h (adjusted odds ratio 2.2 [1.4-3.4], P = 0.001).	Consistent with the anecdotal experience of Australian ED clinicians, the present study demonstrated an increased ED LOS for patients managed in isolation. Enhanced infection prevention and control precautions will be required during and beyond the current pandemic, creating significant ongoing challenges for emergency care systems.
**Australia and New Zealand Emergency Department Airway Registry (ANZEDAR)** *Fogg, 2016 [60]*			
Case series and review of emergency front-of-neck surgical airways from The Australian and New Zealand Emergency Department Airway Registry. Alkhouri et al., 2021.	To describe the management of cases resulting in an eFONA, and recorded in The Australian and New Zealand Emergency Department Airway Registry (ANZEDAR).	An eFONA/CICO rescue airway was performed on 15 adult patients: 14 cricothyroidotomies (0.3% of registry intubations) and one tracheostomy. The indication for intubation was 60% trauma and 40% medical aetiologies. The intubator specialty was emergency medicine in eight (53.3%) episodes. Thirteen (86.7%) cricothyroidotomies and the sole tracheostomy (6.7%) were performed at major referral hospitals with 12 (80%) surgical airways out of hours. In four (26.7%) cases, cricothyroidotomy was performed as the primary intubation method. Preoxygenation techniques were used in 14 (93.3%) episodes; apneic oxygenation in four (26.7%).	Most cases demonstrated deviations from standard practice which may have increased the likelihood of performance of a surgical airways, and its increased likelihood out of hours. Our findings may inform training strategies to improve care for ED patients requiring this critical intervention. We recommend further discussion of proposed standard terminology for emergency surgical or percutaneous airways, to facilitate clear crisis communication.
Ketamine use for rapid sequence intubation in Australian and New Zealand emergency departments from 2010 to 2015: A registry study. Ferguson et al., 2019.	To describe the incidence of ketamine use in Australasian EDs contributing to the Australian and New Zealand Emergency Department Airway Registry (ANZEDAR) from 2010 to 2015, and to evaluate whether there has been any increase in ketamine use. We also aimed to identify any predictors of ketamine use.	A total of 4658 patients met inclusion criteria. The annual incidence of ketamine use increased from 5% to 28% over the study period (P < 0.0001). In the logistic regression analysis, the presence of an emergency physician as a team leader was the strongest predictor of ketamine use (odds ratio [OR] 1.83, 95% confidence interval [CI] 1.44-2.34). The OR for an increase in one point on the Glasgow Coma Scale was 1.10 (95% CI 1.07-1.12), whereas an increase of 1 mmHg of systolic blood pressure had an OR of 0.98 (95% CI 0.98-0.99). Intubation occurring in a major referral hospital had an OR of 0.68 (95% CI 0.56-0.82), while trauma conferred an OR of 1.38 (95% CI 1.25-1.53).	Ketamine use increased between 2010 and 2015. Lower systolic blood pressure, the presence of an emergency medicine team leader, trauma and a higher Glasgow Coma Scale were associated with increased odds of ketamine use. Intubation occurring in a major referral center was associated with lower odds of ketamine use.

Bed tilt and ramp positions are associated with increased first-pass success of adult endotracheal intubation in the emergency department: A registry study. Bennett et al., 2023.	To identify current patient positioning practices during endotracheal intubation and its association with first-pass success at intubation and adverse event rates in obese and non-obese populations.	A total of 3708 intubations from 43 EDs were included. Overall, the non-obese cohort had a greater FPS rate (85.9%) compared to the obese group (77.0%). The bed tilt position had the highest FPS rate (87.2%), whereas the supine position had the lowest (83.0%). AE rates were highest in the ramp position (31.2%) compared to all other positions (23.8%). Regression analysis showed ramp, or bed tilt positions and a consultant-level intubator were associated with higher FPS. Obesity, in addition to other factors, was independently associated with lower FPS.	Obesity was associated with lower FPS, which could be improved through performing a bed tilt or ramp positioning.
**Korean Emergency Airway Management Registry (KEAMR)** *Choi, 2012 [63]*			
Current status of emergency airway management for elderly patients in Korea: Multicenter study using the Korean Emergency Airway Management Registry. Cho et al., 2013.	To report the current status and complications of emergency airway management in Korean elderly patients by analyzing data from a multicenter registry database.	The study consisted of 4891 patients aged 65 years and older. The anticipation rate of difficult airway for age younger than 65 years, age 65 years and older and age 80 years and older was 15.8% (805/5090; 95% confidence interval [CI], 14.8-16.8), 12.0% (376/3146; 95% CI, 10.9-13.1) and 8.5% (97/1140; 95% CI, 7.0-10.0) respectively (P < 0.001). Rapid sequence intubation was performed on 27.8% (1683/6051; 95% CI, 26.7-29.0) of the patients aged younger than 65 years, 26.5% (954/3599; 95% CI, 25.1-28.0) of the patients aged between 65 and 79 years and 25.2% (325/1292; 95% CI, 22.8-27.6) of the patients aged 80 years and older (P = 0.097). First pass success was shown in 4837 (79.9%; 95% CI, 78.9-80.9) of 6051 patients aged less than 65 years, 2868 (79.7%; 95% CI, 78.3-81.0) of 3599 patients aged between 65 and 79 years and 1069 (82.7%; 95% CI, 80.5-84.7) of 1292 patients aged 80 years and older (P = 0.047). No differences were found between each group in the comparison of complication incidence by age (P = 0.686).	Similar success and complication rates were found for emergency airway management in elderly patients compared with patients aged less than 65 years.
The clinical effectiveness of simulation based airway management education using the Korean emergency airway registry. Kim et al, 2017.	To evaluate the effectiveness of a simulation based emergency airway management program (SBEAMP) in actual practice.	The ratio of patients with no medicine received during intubation showed a decrease in both groups but was more rapid in the participant group (p<0.001). The ratio of intubation with sedatives alone was high in the non-participant group (P<0.001). The ratio of intubation with paralytics alone was high in the non-participant group (p<0.001). In the participant group, a combination of both agents was used more frequently (P<0.001). Cases of intubation with both agents and preoxygenation were more prevalent in the participant group (P<0.001).	We concluded in this study that simulation based emergency airway management program (SBEAMP) had a positive influence on actual clinical outcomes in emergency airway management.
**National Emergency Airway Registry (NEAR)** *Brown, 2015 [49]*			
Techniques and Trends, Success Rates, and Adverse Events in Emergency Department Pediatric Intubations: A Report From the National Emergency Airway Registry. Pallin et al., 2016.	To describe ED intubation practices for children younger than 16 years.	Ten of 18 participating centers provided qualifying data, reporting 1,053 encounters. Emergency physicians initiated 85% of intubations. Trainees initiated 83% (95% confidence interval [CI] 81% to 85%). Premedication became uncommon, reaching less than 30% by the last year. Etomidate was used in 78% of rapid sequence intubations. Rocuronium use increased during the period of study, whereas succinylcholine use declined. Video laryngoscopy increased, whereas direct laryngoscopy declined. The first attempt was successful in 83% of patients (95% CI 81% to 85%) overall. The risk of first-attempt failure was highest for infants (relative risk versus all others 2.3; 95% CI 1.8 to 3.0). Odds of first-attempt success for girls relative to boys were 0.57.	Because we sampled only 10 centers and most of the intubations were by trainees, our results may not be generalizable to the typical ED setting. We found that premedication is now uncommon, etomidate is the predominant induction agent, and rocuronium and video laryngoscopy are used increasingly. First- attempt failure is most common in infants.

		The odds were 3.4 times greater for rapid sequence intubation than sedation without paralysis. The ultimate success rate was 99.5%.	
A 12-month descriptive analysis of emergency intubations at Brooke Army Medical Center: a National Emergency Airway Registry study. April et al., 2017.	To describe the intubation experiences, practices, and outcomes of the active duty physicians at Brooke Army Medical Center (BAMC) over a 12-month period.	During the study period, providers performed 259 intubations in the BAMC ED. Reasons for intubation were related to trauma for 184 patients (71.0%) and medical conditions for 75 patients (29.0%). Overall, first-attempt success was 83.0%. Emergency medicine residents performed a majority of first attempts (95.0%). Most common devices chosen on first attempt were a video laryngoscope for 143 patients (55.2%) and a direct laryngoscope for 115 patients (44.4%). One patient underwent cricothyrotomy. The 2 most common induction agents were ketamine (59.8%; 95% CI, 55.2%- 67.4%) and etomidate (19.3%; 95% CI, 14.7%-24.7%). The most common neuromuscular blocking agents were rocuronium (62.9%; 95% CI, 56.7%- 68.8%) and succinylcholine (18.9%; 95% CI, 14.3%-24.2%).	In the BAMC ED, emergency intubation most commonly occurred for trauma indications using video laryngoscopy with a high first-pass success.
Factors associated with post-intubation sedation after emergency department intubation: A Report from The National Emergency Airway Registry. Lembersky et al., 2020.	To examine the rates of post-intubation sedation and identify associated factors utilizing this multicentered dataset in order to better identify patient populations who may be at risk from lack of post-intubation sedation.	Of the 11,748 eligible intubations, 9099 received post-intubation sedation (77.5%) while 2649 did not (22.5%). Pre-intubation hypotension (odds ratio; 95% confidence Interval) (0.27; 0.24-0.31) and post-intubation hypotension (0.27; 0.24-0.31) were associated with lower odds of post-intubation sedation. Patients with a medical indication compared to a traumatic indication for ETI had higher odds of receiving post-intubation sedation (1.16; 1.05-1.28) as did those that underwent rapid sequence intubation (15.15; 13.56-16.93). Use of succinylcholine was associated with a higher odd of post-intubation sedation compared to a long-acting neuro-muscular blocking agent (i.e. rocuronium or vecuronium) (1.89; 1.68-2.12).	Post-intubation sedation rates in NEAR are higher than previously reported and multiple factors including the indication for intubation and succinylcholine use, are associated with higher odds of receiving post-intubation sedation.
Telemedicine-Assisted Intubation in Rural Emergency Departments: A National Emergency Airway Registry Study. VanOeveren et al., 2017.	To describe telemedicine-assisted intubation in rural EDs that are served by a large ED telemedicine network in the upper Midwest. Secondary objectives include comparing the success between intubations performed by direct laryngoscopy (DL) and VL, reporting the frequency of interventions using telemedicine consultation, and reporting complications and clinical outcomes.	Included were 206 intubations. The most common indication for intubation was respiratory failure. First-pass success rate (post activation) was 71%, and 96% were eventually intubated. Most attempts (66%) used rapid-sequence intubation. Fifty-four percent of first attempts used video laryngoscopy (VL). Telemedicine providers intervened in 24%,43%, and 55% of first-third attempts, respectively. First-pass success with VL and direct laryngoscopy was equivalent (70% vs. 71%, p = 0.802). Adverse events were reported in 49 cases (24%), which were most frequently hypoxemia.	The impact of telemedicine during emergency intubation is not defined. We showed a 71% first-pass rate post-telemedicine linkage (70% of cases had a previous attempt). Our ultimate success rate was 96%, similar to that in large-center studies. Telemedicine support may contribute to success. Telemedicine-supported endotracheal intubation performed in rural hospitals is feasible, with good success rates. Future research is required to better define the impact of telemedicine providers on emergency airway management.
Multicenter Comparison of Nonsupine Versus Supine Positioning During Intubation in the Emergency Department: A National Emergency Airway Registry (NEAR) Study. Stoecklein et al., 2019.	To compare the rate of first-pass success, peri-intubation adverse events, and Cormack and Lehane laryngeal view for patients undergoing intubation in supine position (SP) and non-supine position (NSP) across multiple EDs.	Of 11,480 total intubations, 5.8% were performed in NSP. The NSP group included significantly more obese patients (OR = 2.2 [95% CI = 1.9-2.6]) and patients with a suspected difficult airway (OR = 1.8 [95% CI = 1.6-2.2]). First-pass success (adjusted OR = 1.1 [95% CI = 0.9-1.4]) and overall rate of grade I glottic views (OR = 1.1 [95% CI = 0.9-1.2]) were similar between groups while NSP had a significantly higher rate of grade I views when direct laryngoscopy was employed (OR = 1.27 [95% CI = 1.04-1.54]). NSP was associated with higher odds of any adverse event (OR = 1.4 [95% CI = 1.1-1.7]).	ED providers utilized SP in most ED intubations but were more likely to use NSP for patients who were obese or in whom they predicted a difficult airway. We found no differences in first-pass success between groups but total adverse events were more likely in NSP. A randomized trial comparing patient positioning during intubation in the ED is warranted.

Incidence of rescue surgical airways after attempted orotracheal intubation in the emergency department: A National Emergency Airway Registry (NEAR) Study. Offenbacher et al., 2023.	To describe the incidence of rescue surgical airways in the ED and characterize the indications for the intervention using the most current iteration of the National Emergency Airway Registry (NEAR).	Of 19,071 subjects in NEAR, 17,720 (92.9%) were ≥14 years old with at least one initial orotracheal or nasotracheal intubation attempt, 49 received a rescue surgical airway attempt, an incidence of 2.8 cases per 1000 (0.28% [95% confidence interval 0.21 to 0.37]). The median number of airway attempts prior to rescue surgical airways was 2 (interquartile range 1, 2). Twenty-five were in trauma victims (51.0% [36.5 to 65.4]), with neck trauma being the most common traumatic indication (n = 7, 14.3% [6.4 to 27.9]).	Rescue surgical airways occurred infrequently in the ED (0.28% [0.21 to 0.37]), with approximately half performed due to a trauma indication. These results may have implications for surgical airway skill acquisition, maintenance, and experience.
Video Laryngoscopy Compared to Augmented Direct Laryngoscopy in Adult Emergency Department Tracheal Intubations: A National Emergency Airway Registry (NEAR) Study. Brown et al., 2020.	To compare first-attempt intubation success using direct laryngoscopy augmented by laryngeal manipulation, ramped patient positioning, and use of a bougie (A-DL) with unaided video laryngoscopy (VL) in adult emergency department (ED) intubations.	We analyzed 11,714 intubations performed from January 1, 2016, through December 31, 2017. Of these encounters, 6,938 underwent orotracheal intubation with either A-DL or unaided VL on first attempt. A-DL was used first in 3,936 (56.7%, 95% CI = 46.9 to 66.5) versus unaided VL in 3,002 (43.3%, 95% CI = 33.5 to 53.1). Of the A-DL first intubations 1,787 (45.4%) employed ramped positioning alone, 1,472 (37.4%) had external laryngeal manipulation (ELM), and 365 (9.3%) used a bougie. Rapid sequence intubation (RSI) was the most common method used in 5,602 (80.8%, 95% CI = 77.0 to 84.5) cases. First-attempt success was significantly higher with all VL (90.9%, 95% CI = 88.7 to 93.1) versus all A-DL (81.1%, 95% CI = 78.7 to 83.5) despite the VL group having more patients with reduced mouth opening, neck immobility, and an initial impression of airway difficult. Multivariable regression analyses controlling for indication, method, operator specialty and year of training, center clustering, and all registry-recorded difficult airway predictors revealed first-attempt success was higher with all unaided VL compared with any A-DL (adjusted OR [AOR] = 2.8, 95% CI = 2.4 to 3.3), DL with bougie (AOR = 2.7, 95% CI = 2.1 to 3.5), DL with ELM (AOR = 1.8, 95% CI = 1.5 to 2.2), DL with ramped positioning (AOR = 2.8, 95% CI = 2.3 to 3.3), or DL with ELM plus bougie (AOR = 2.8, 95% CI = 2.3 to 3.3). Subgroup analyses of HA-VL and SG-VL compared with any A-DL yielded similar results (AOR = 3.2, 95% CI =2.6 to 3.0; and AOR = 2.4, 95% CI = 1.9 to 3.0, respectively). The propensity score-adjusted odds for first-attempt success with VL was also 2.8 (95% CI = 2.4 to 3.3). Fewer esophageal intubations were observed in the VL cohort (0.4% vs. 1.3%, AOR = 0.2, 95% CI = 0.1 to 0.5).	Video laryngoscopy used without any augmenting maneuver, device, or technique results in higher first-attempt success than does DL that is augmented by use of a bougie, ELM, ramping, or combinations thereof.
Extraglottic device use is rare during emergency airway management: A National Emergency Airway Registry (NEAR) study. April et al., 2023.	To describe the patient, operator, and procedural characteristics of Extraglottic device (EGD) use during airway management using a multi-center registry of ED intubations.	Of 19,071 patients undergoing intubation attempts, 56 (0.3%) underwent EGD placement. Of 25 participating sites, 13 reported no cases undergoing EGD placement; the median number of EGDs placed per site was 2 (interquartile range 1-2.5, range 1-31). Twenty-nine (54%) patients had either hypotension or hypoxia prior to the start of airway management. Clinicians reported anticipation of a difficult airway in 55% and at least one difficult airway characteristic in 93% of these patients. Forty-one encounters entailed placement of a laryngeal mask airway (LMAÂ®) Fastrach™, 33 of whom underwent subsequent successful intubation through the EGD and 7 of whom underwent intubation by alternative methods. An additional 10 encounters utilized a standard LMAÂ® device. Providers placed 34 (61%) EGDs during the first intubation attempt. Seventeen EGD patients (30%) experienced peri-	EGD use was rare in this multi-center ED registry. EGD occurred predominantly in patients with difficult airway characteristics with favorable airway management outcomes. Clinicians should consider this emergency airway device for patients with a suspected difficult airway.

		procedure adverse events, including 14 (25%) experiencing hypoxemia. None of these patients expired due to failed airways.	
Video Laryngoscopy Is Associated With First-Pass Success in Emergency Department Intubations for Trauma Patients: A Propensity Score Matched Analysis of the National Emergency Airway Registry. Trent et al., 2021.	To (1) characterize endotracheal intubations in trauma patients in US EDs and estimate (2) first-pass success and (3) the associations between patient and intubation characteristics and first-pass success	Of the 19,071 intubations in NEAR, 4,449 (23%) were for trauma, and nearly all (88%) had at least one difficult airway characteristic. Prevalence of first-pass success was 86.8% (95% confidence interval [CI]: 83.3% to 90.3%). Most patients were intubated with video laryngoscopy, and patients were more likely to be intubated on first-pass with video laryngoscopy as compared to direct laryngoscopy (90% versus 79%). After propensity score matching, video laryngoscopy remained associated with first-pass success (adjusted risk difference 11%, 95% CI: 8% to 14%; and OR 2.2, 95% CI: 1.6 to 2.9). Additionally, an initial impression of difficult airway, blood/vomit in the airway, and use of external laryngeal manipulation were all associated with decreased odds of first-pass success.	Emergency physicians are successful at intubating patients in the setting of trauma, and video laryngoscopy is associated with twice the odds of first-pass success when compared to direct laryngoscopy.
Peri-intubation cardiac arrest in the Emergency Department: A National Emergency Airway Registry (NEAR) study. April et al., 2021.	To determine the incidence of peri-intubation cardiac arrest through analysis of a multi-center Emergency Department (ED) airway registry and to report associated clinical characteristics	Of 15,776 subjects who met selection criteria, 157 (1.0%, 95% CI 0.9 1.2%) experienced peri-intubation cardiac arrest. Pre-intubation systolic blood pressure <100 mm Hg (aOR 6.2, 95% CI 2.5 8.5), pre-intubation oxygen saturation <90% (aOR 3.1, 95% CI 2.0 4.8), and clinician-reported need for immediate intubation without time for full preparation (aOR 1.8, 95% CI, 1.2 2.7) were associated with higher likelihood of peri-intubation cardiac arrest. The association between pre-intubation shock and cardiac arrest persisted in additional modeling stratified by ETI indication, induction agent, and oxygenation status.	Peri-intubation cardiac arrest for patients undergoing ETI in the ED is rare. Higher likelihood of arrest occurs in patients with pre-intubation shock or hypoxemia. Prospective trials are necessary to determine whether a protocol to optimize pre-intubation hemodynamics and oxygenation mitigates the risk of peri-intubation cardiac arrest.
First-Attempt Success Between Anatomically and Physiologically Difficult Airways in the National Emergency Airway Registry. Nikolla et al., 2024.	To examine the difference in first-attempt success between anticipated difficult airways (ADAs alone, PDAs alone, and combined ADAs and PDAs) and those without difficult airway characteristics (neither ADAs nor PDAs) using a multicenter registry of ED intubations.	Of the 19,071 subjects intubated during the study period, 13,938 were included in the study. Compared to those without difficult airway findings (neither ADA nor PDA), the adjusted odds ratios (aORs) for first-attempt success were 0.53 (95% CI, 0.40-0.68) for ADAs alone, 0.96 (0.68-1.36) for PDAs alone, and 0.44 (0.34-0.56) for both. The aORs for first-attempt success without adverse events were 0.72 (95% CI, 0.59-0.89) for ADAs alone, 0.79 (0.62-1.01) for PDAs alone, and 0.44 (0.37-0.54) for both. There was no evidence that the interaction between ADAs and PDAs for first-attempt success with or without adverse events was different from additive (ie, not synergistic/multiplicative or antagonistic).	Compared to no difficult airway characteristics, ADAs were inversely associated with first-attempt success, while PDAs were not. Both ADAs and PDAs, as well as their interaction, were inversely associated with first-attempt success without adverse events.
Risk of peri-intubation adverse events during emergency department intubation of overdose patients: a national emergency airway registry (near) analysis. Kunzler et al., 2022.	To quantify the frequency of peri-intubation adverse events for patients intubated for overdose and to determine whether first attempt success and first attempt success without adverse events differs between patients intubated for overdose and patients for other reasons.	We analyzed 17,984 patients, including 1,983 (11%) intubated for overdose, and 16,001 (89%) intubated for other indications. Patients intubated for overdose were younger (median age 38 vs 55 years), were less frequently obese (26% vs 34%), and fewer had difficult airway characteristics (38% vs 53%). Overdose patients were more likely to have preoxygenation performed (45% vs 35%), more likely to have apneic oxygenation (39% vs 31%), and more likely to have bougie used (33% vs 17%). First attempt success was 90.5% in patients intubated for overdose and 87.5% in patients intubated for other reasons (absolute difference 3.0%; 95% CI: 1.3 to 7.3). First attempt success without adverse events was higher in overdose patients (85.0%) compared to other patients (78.7%) (absolute difference, 6.3%; 95% CI 1.0 to 11.7%). Overdose	For patients in whom the primary indication for intubation is overdose there is an increased chance of first attempt success without adverse event.

		patients experienced significantly less hypotension (1.5% vs 4.1%), and tended to have fewer adverse events overall. Multivariable model results were consistent with the unadjusted results including no difference in first pass success (adjusted odd ratio 1.02 [95% CI 0.86-1.23]). There was a higher first pass success without complication in patients intubated for overdose (adjusted odds ratio 1.23; 95% CI 1.07 to 1.43).	
Emergency Department Intubation Success With Succinylcholine Versus Rocuronium: A National Emergency Airway Registry Study. April et al., 2018.	To compare first-pass intubation success and peri-intubation adverse events between rapid sequence intubation performed with succinylcholine versus rocuronium.	There were 2,275 rapid sequence intubations facilitated by succinylcholine and 1,800 by rocuronium. Patients receiving succinylcholine were younger and more likely to undergo intubation with video laryngoscopy and by more experienced providers. First-pass intubation success rate was 87.0% with succinylcholine versus 87.5% with rocuronium (adjusted odds ratio 0.9; 95% confidence interval 0.6 to 1.3). The incidence of any adverse event was also comparable between these agents: 14.7% for succinylcholine versus 14.8% for rocuronium (adjusted odds ratio 1.1; 95% confidence interval 0.9 to 1.3). We observed similar results when they were stratified by paralytic weight-based dose.	In this large observational series, we did not detect an association between paralytic choice and first-pass rapid sequence intubation success or peri-intubation adverse events.
Direct vs Video Laryngoscopy for Difficult Airway Patients in the Emergency Department: A National Emergency Airway Registry Study. Ruderman et al., 2022.	To measure the rates of FPS comparing VL vs DL intubations in adult ED patients who had an anticipated or identified anatomically difficult airway. Also sought to answer the question of whether VL design (hyperangulated vs standard geometry) influenced FPS in these patients. Secondary goal was to determine whether there were differences in peri-intubation adverse events between these two intubation methods.	Of 18,123 total intubations, 12,853 had a predicted or identified anatomically difficult airway. The FPS for difficult airways was 89.1% (95% CI 85.9-92.3) with VL and 77.7% (95% CI 75.7-79.7) with DL (P <0.00001). The FPS rates were similar between VL subtypes for all difficult airway characteristics except airways with blood or vomit, where SGVL FPS (87.3%; 95% CI 85.8-88.8) was slightly better than HAVL FPS (82.4%; 95% CI, 80.3-84.4). Adverse event rates were similar except for esophageal intubations and vomiting, which were both less common in VL than DL. Esophageal intubations occurred in 0.4% (95% CI 0.1-0.7) of VL attempts and 1.5% (95% CI 1.1-1.9) of DL attempts. Vomiting occurred in 0.6% (95% CI 0.5-0.7) of VL attempts and 1.4% (95% CI 0.9-1.9) of DL attempts.	Analysis of the NEAR database demonstrates higher first-pass success with VL compared to DL in patients with predicted or anatomically difficult airways, and reduced rate of esophageal intubations and vomiting.
Awake intubations in the emergency department: A report from the National Emergency Airway Registry. Kaisler et al., 2021.	To describe awake intubation practices in the emergency department (ED) and report success, complications, devices used, and rescue techniques using multicenter surveillance.	Of 19,071 discrete patient encounters, an awake technique was used on the first attempt in 82 (0.4%) patients. The majority (91%) of first attempts were performed by emergency medicine physicians. Angioedema (32%) and non-angioedema airway obstruction (31%) were the most common indications for an awake intubation attempt. The most common initial device used was a flexible endoscope (78%). Among all awake intubations first-attempt success was achieved in 85% (95% CI [76%-95%]), and peri-intubation complications occurred in 16% (95% CI [9%-26%]).	Awake intubation in this multicenter cohort of emergency department patients was rare and was performed most often in patients with airway edema or obstruction. Emergency physicians performed the majority of first intubation attempts with high first-attempt success. Further studies are needed to determine optimal emergency airway management in this patient population.
First-Attempt Intubation Success Among Emergency Medicine Trainees by Laryngoscopic Device and Training Year: A National Emergency Airway Registry Study. Garcia et al., 2023.	To compare intubation first-attempt success with the direct laryngoscope, hyperangulated video laryngoscope, and standard geometry video laryngoscope among emergency medicine residents at various postgraduate years (PGY) of training.	Among 15,204 intubations performed by emergency medicine trainees, first-attempt success for PGY-1, PGY-2, and PGY3+ residents, respectively were: 78.8% (95% CI, 75.0 to 82.2%), 81.3% (79.4 to 83.0), and 83.6% (95% CI, 82.1 to 85.1) for direct laryngoscope; 87.2% (95% CI, 84.2 to 89.7), 90.4% (95% CI, 88.8 to 91.9%), and 91.2% (95% CI, 89.8 to 92.5%) for hyperangulated video laryngoscope; and 88.7% (95% CI, 86.1 to 90.9), 90.2% (95% CI, 88.7 to 91.5%), and 94.6% (95% CI 93.9 to 95.3%) for standard geometry video laryngoscope. Direct laryngoscope first-attempt success improved for PGY-2 (adjusted odds	Each laryngoscopy device class was associated with improvement in first-attempt success as training progressed. The video laryngoscope outperformed the direct laryngoscope for all operator groups, and PGY-1 trainees achieved higher first attempt success using a standard geometry video laryngoscope than PGY-3+ trainees using a direct laryngoscope. These

		ratio [aOR],1.41; 95% CI, 1.09 to 1.82) and PGY-3+ (aOR, 1.76; 1.36 to 2.27) trainees compared to PGY-1. Hyperangulated video laryngoscope success also improved for PGY-2 (aOR, 1.51; 1.1 to 2.05) and PGY-3+ (aOR, 1.56; 1.15 to 2.13) trainees compared to PGY-1. For the standard geometry video laryngoscope, only PGY-3+ (aOR, 1.72; 1.25 to 2.36) was associated with improved first-attempt success compared to PGY-1.	findings support the conjecture that in adult patients, a direct laryngoscope should not be routinely used for the first intubation attempt unless clinical circumstances, such as the presence of a soiled airway, would favor its success. These findings need to be validated with prospective randomized clinical trials.
Emergency airway management in a Singapore center: A registry study. Chan et al., 2021.	To describe the intubation indications, prevalence of difficult airway features, peri-intubation adverse events and intubator characteristics in the ED of the National University Hospital, Singapore.	There were 669 patients, with male predominance (67.3%, 450/669) and mean age of 60.9 years (standard deviation [SD] 18.1). Of these, 25.6% were obese or grossly obese and majority were intubated due to medical indications (84.8%, 567/669). Emergency physicians' initial impression of difficult airway correlated with a higher grade of glottis view on laryngoscopy. First-pass intubation success rate was 86.5%, with hypoxia (11.2%, 75/669) and hypotension (3.7%, 25/669) reported as the two most common adverse events. Majority was rapid sequence intubation (67.3%, 450/669) and the device used was most frequently a video laryngoscope (75.6%, 506/669). More than half of the intubations were performed by postgraduate clinicians in year 5 and above, clinical fellows or attending physicians.	In our center, the majority of emergency intubations were performed for medical indications by senior doctors utilizing rapid sequence intubation and video laryngoscopy with good first-attempt success.
Emergency Department Intubations in Patients With Angioedema: A Report from the National Emergency Airway Registry. Sandefur et al., 2021.	To describe patient characteristics, emergency airway management techniques, and outcomes among patients in the ED who were enrolled in a multicenter prospective registry and were managed for angioedema.	Of 19,071 patient encounters, intubation was performed for angioedema in 98 (0.5%). First-attempt success was achieved in 81%, with emergency physicians performing the procedure in 94% of encounters. The most common device used was a flexible endoscope (49%), and 42% of attempts were via a nasal route. Pharmacologic methods included sedation with paralysis (61%), topical anesthesia with or without sedation (13% and 13%, respectively), and sedation only (10%). Among 19 (19%) patients requiring additional attempts, intubation was achieved on second attempt in 10 (53%). The most common adverse events were hypotension (13%) and hypoxemia (12%). Cricothyrotomy occurred in 2 patients (2%). No deaths were observed.	Angioedema was a rare indication for intubation in the ED setting. Emergency physicians achieved first-attempt success in 81% of encounters and used a broad range of intubation devices and methods, including flexible endoscopic techniques. Cricothyrotomy was rare, and no ED deaths were reported.
Ketamine Versus Etomidate and Peri-intubation Hypotension: A National Emergency Airway Registry Study. April et al., 2020.	To compare patient outcomes between intubations performed with ketamine vs etomidate using a large prospective observational multicenter ED cohort.	There were 738 encounters with ketamine and 6,068 with etomidate. Patients receiving ketamine were more likely to have difficult airway characteristics (effect size difference = 8.8%, 95% confidence interval [CI] = 5.3% to 12.4%) and to undergo intubation with video laryngoscopy (8.1%, 95% CI = 4.4% to 12.0%). Peri-intubation hypotension incidence was 18.3% among patients receiving ketamine and 12.4% among patients receiving etomidate (effect size difference = 5.9%, 95% CI = 2.9% to 8.8%). Patients receiving ketamine were more likely to receive treatment for peri-intubation hypotension (effect size difference = 6.5%, 95% CI = 3.9% to 9.3%). In logistic regression analyses, patients receiving ketamine remained at higher risk for peri-intubation hypotension (aOR = 1.4, 95% CI = 1.2 to 1.7) and treatment for hypotension (aOR = 1.8, 95% CI = 1.4 to 2.0). There was no difference in the aOR of hypotension between patients receiving ketamine at doses ≤1.0 mg/kg versus >1.0 mg/kg or patients receiving etomidate at doses ≤0.3 mg/kg versus >0.3 mg/kg.	Pending additional data, our results suggest that clinicians should not necessarily prioritize ketamine over etomidate based on concern for hemodynamic compromise among ED patients undergoing intubation.
**Emergency Medicine Pulmonary Embolism in the Real World Registry (EMPEROR)** *Pollack, 2011 [33]*			

Therapy and outcomes in massive pulmonary embolism from the Emergency Medicine Pulmonary Embolism in the Real World Registry. Lin et al., 2012.	To characterize the use of aggressive therapies in patients with and without MPE and from the Emergency Medicine Pulmonary Embolism in the Real World (EMPEROR) and compare outcomes (major hemorrhage, recurrent VTE, and death) in patients with vs without MPE.	Of 1875 patients enrolled, 58 (3.1%) had MPE. There was no difference in frequency of parenteral anticoagulation (98.3% [95% confidence interval {CI}, 90.5-101.6] vs 98.5% [95% CI, 97.9-99.1], P = .902) between patients with and without MPE. Fibrinolytic therapy and embolectomy were infrequently used but were used more in patients with MPE than in patients without MPE (12.1% [95% CI, 3.7-20.5] vs 2.4% [95% CI, 1.7-3.1], P b .001, and 3.4% [95% CI, 0.0-8.1] vs 0.7% [95% CI, 0.3-1.1], P = .022, respectively). Comparison of outcomes revealed higher all-cause inpatient mortality (13.8% [95% CI, 4.9-22.7] vs 3.0% [95% CI, 2.2-3.8], P b .001), higher risk of inpatient bleeding complications (10.3% [95% CI, 2.5-18.1] vs 3.5% [95% CI, 2.7-4.3], P = .007), and a higher 30-day mortality (14.0% [95% CI, 4.4-23.6] vs 1.8% [95% CI, 1.2-2.4], P b .001) for patients with MPE.	In a contemporary registry of ED patients, MPE mortality was 4-fold higher than patients without MPE, yet only 12% of the MPE cohort received fibrinolytic therapy. Variability exists between the treatment of MPE and current recommendations.
**Risk Profile of Patients VTED Attended in Spanish Emergency Departments Registry (ESPHERIA)** *Jimenez, 2017 [53]*			
Clinical characteristics and course in emergency department patients with chronic obstructive pulmonary disease and symptomatic acute venous thromboembolic disease: secondary analysis of the ESPHERIA registry.	To determine the impact of chronic obstructive pulmonary disease (COPD) on prognosis in patients diagnosed with venous thromboembolic disease (VTED) in Spanish emergency departments.	A total of 801 patients, 71 (9%) with COPD, were included. Pulmonary thromboembolism was recorded in 77.%% of the patients with COPD (vs in 47.1% of patients without COPD; P<.001). Patients with COPD had evidence of right ventricular dysfunction on computed tomography angiography more often than other VTED patients (18.2% vs 13.1%; P<.001) and more often required ventilatory support (7% vs 0.5%; P<.001). VTED patients with COPD also had a higher rate of readmission or mortality at 180 days (hazard ratio, 1.52; 95% CI, 1.00-2.29; P = .048)] than patients without COPD.	COPD affects the prognosis of patients diagnosed with VTED in Spanish emergency departments as evidenced by hospital readmission and mortality.
**Emergency Atrial Fibrillation Registry of the Catalan Institute of Health (URGFAICS)** *Jacob 2019* [55]			
Impact of emergency department management of atrial fibrillation with amiodarone on length of stay. A propensity score analysis based on the URGFAICS registry. Cabello, et al., 2020.	To compare the length of ED stay of atrial fibrillation patients who were treated with or without amiodarone.	Of the 1199 patients included in the registry, 225 patients (18.8%) were treated with amiodarone while 974 (81.2%) were not. We performed a univariate study depending on amiodarone administration followed by propensity score calculation according to the 14 statistically different features found previously and six significant variables, obtaining 150 patients (75 for each group) suitable for the analysis. The length of ED stay was analyzed using box plot, with a P <0.001 in the crude analysis and P=0.012 after propensity score matching and using survival curves for the analysis of prolonged ED stay, with a log rank <0.001 in the crude analysis and log rank 0.021 after the propensity score-matched analysis.	Amiodarone is associated with longer length of ED stay until discharge independently of the baseline characteristics of the patients.
**Epidemiology of Acute Heart Failure in Emergency Departments (EAHFE) Registry** *Llorens, 2015* [45]			
Effects on short term outcome of non-invasive ventilation use in the emergency department to treat patients with acute heart failure: A propensity score-based analysis of the EAHFE Registry. Miró et al., 2018.	To assess the effects on short-term outcome of the use of non-invasive ventilation (NIV) in the ED setting to treat patients diagnosed with acute heart failure (AHF).	Of 11,152 patients from the EAHFE (age (SD): 80 (10) years; 55.5% women), 718 (6.4%) were NIV+ and had a higher 30-day mortality (HR = 2.229; 95%CI = 1.861-2.670) (p < 0.001). PS matching provided 2 groups of 490 patients each with no significant differences in 30-day mortality (HR = 1.239; 95%CI = 0.905-1.696) (p = 0.182). Interaction analysis suggested a worse effect of NIV on elderly patients (> 85 years, p < 0.001), AHF associated with ACS (p = 0.045),	The use of NIV to treat AHF in ED is not associated with improved mortality outcomes and should be cautious in old patients and those with ACS and hypotension.

		and SBP < 100 mmHg (p < 0.001). No significant differences were found in the secondary endpoints except for more prolonged hospitalizations in NIV+ patients (OR = 1.445; 95%CI = 1.122-1.862) (p = 0.004).	
Early intravenous nitroglycerin use in prehospital setting and in the emergency department to treat patients with acute heart failure: Insights from the EAHFE Spanish registry. Miró et al., 2021.	To explore the effects of prehospital administration of nitrates on mortality and adverse events in the EAHFE (Epidemiology of AHF in Emergency departments) Spanish registry that includes patients diagnosed with AHF in emergency department (ED) consecutively included in sets of 1-2 months cohorts during last 15 years. In addition, we have also explored the effects of IV NTG when this is started in the ED.	We included 8424 patients: preED-NTG = 292 (3.5%), ED-NTG = 1159 (13.8%) and no-NTG = 6973 (82.7%). preED-NTG group had the most severely decompensated cases of AHF (p < 0.001) but it had lower in-hospital (OR = 0.724, 95%CI = 0.459-1.114), 30-day (HR = 0.818, 0.576-1.163) and 365-day mortality (HR =0.692, 0.551-0.869) and 90-day post-discharge events (HR = 0.795, 0.643-0.984) than control group. ED-NTG group had mortalities similar to control group (in-hospital: OR = 1.164, 0.936-1.448; 30-day: HR = 0.980, 0.819-1.174; 365-day: HR = 0.929, 0.830-1.039) but significantly decreased 90-day post-discharge events (HR = 0.870, 0.780-0.970). Prolonged hospitalization rate did not differ among groups. Five different analyses confirmed these findings.	Early prehospital IV NTG administration was associated with lower mortality and post-discharge events, while IV NTG initiated in ED only improved post-discharge event rate. Further studies are needed to assess the role of early prehospital administration of IV NTG to patients with AHF.
**Procedural Sedation in the Community Emergency Department Registry (ProSCED)** *Sacchetti, 2007* [30]			
Pediatric Procedural Sedation in the Community Emergency Department: results from the ProSCED registry.	This report examines the pediatric findings of the ProSCED registry and identifies the characteristics of children requiring procedural sedation along with the practice preferences of their attending EPs in the community hospital setting.	A total of 1028 procedural sedations were performed on 977 patients at 14 study sites, with 341 procedures performed in 339 patients younger than 21 years. The most common specified pediatric procedures performed included laceration repairs (n = 86, 25%), shoulder relocations (n = 78, 23%), and fracture care of the upper extremity (n = 56, 16%). Medications used included ketamine (n = 141, 41%), midazolam (n = 10, 32%), etomidate (n = 54, 16%), fentanyl (n = 51, 15%), and propofol (n = 47, 14%). Complications were reported in 2 cases (0.6%), 1 episode of apnea requiring a reversal agent and 1 episode of hypoxia responsive to supplemental oxygen. Of procedures attempted, 339 (99.4%) were successfully completed. Emergency physicians both directed the sedation and performed the procedure in 252 cases (74%), whereas in another 69 cases (20%), they directed the sedation for another physician performing the procedure. In 20 cases (5.8%), the EP directed sedation for a painless diagnostic study.	Community EPs in the Procedural Sedation in the Community Emergency Department registry deliver safe and effective pediatric sedation using a broad selection of agents.
**VNICat (NIVCat in English)** *Jacob, 2019 [55]*			
Characteristics of prolonged noninvasive ventilation in emergency departments and impact upon effectiveness. Analysis of the VNICat registry.	To analyze the characteristics and variables associated with prolonged times of completely implemented prolonged NIV at the hospital emergency services (HES) setting (NIV-HES). Also, to study the impact this has on the efficacy of the technique.	A total of 125 patients were included, with a median NIV-ED duration of 12 h, which was the cut-off point for the comparator groups. In 60 cases (48%) NIV-ED was not prolonged (<12 h), while in 65 cases (52%) ventilation was prolonged (≥12 h). Non-prolonged NIV-ED was associated to the indication of acute heart failure and prolonged ventilation to the presence of diabetes. There were no differences between non-prolonged and prolonged NIV-ED in terms of efficacy, and the success rate in terms of improvement was 68.3% and 76.9%, respectively, with an adjusted odds ratio of 1.49 (95%CI 0.61-3.60).	Prolonged NIV-ED is a frequent situation, but few variables associated to it have been studied. The presence of prolonged ventilation did not influence the success rate of NIV.
